# Phylogeographic patterns of intertidal arthropods (Acari, Oribatida) from southern Japanese islands reflect paleoclimatic events

**DOI:** 10.1038/s41598-019-55270-z

**Published:** 2019-12-13

**Authors:** Tobias Pfingstl, Maximilian Wagner, Shimpei F. Hiruta, Stephan Koblmüller, Wataru Hagino, Satoshi Shimano

**Affiliations:** 1 Institute of Biology, University of Graz, Universitätsplatz 2, 8010 Graz, Austria; 2 Center for Molecular Biodiversity Research, National Museum of Nature and Science, Amakubo 4-1-1, Tsukuba, Ibaraki, 305-0005 Japan; 3 Department of Bioresource Engineering, National Institute of Technology, Okinawa College, Henoko 905, Nago-City, Okinawa, 905-2192 Japan; 4 Science Research Center, Hosei University, Fujimi 2-17-1 Chiyoda-ku, Tokyo, 102-8160 Japan

**Keywords:** Evolution, Zoology

## Abstract

The Japanese islands represent one of the world’s biodiversity hotspots. Their geological history and present geography resulted in a high number of endemic species in nearly all major metazoan clades. We investigated the phylogeography of three different intertidal mite species from the Ryukyu islands and southern mainland by means of morphometry and molecular genetics. None of the species represents an endemic, nearly all show distributions ranging over at least the southern and central Ryukyus. Two species, *Fortuynia shibai* and *F. churaumi* sp. n. clearly represent sister species that are derived from a common Eastern ancestor. Molecular genetic results indicate that these species separated approx. 3 Ma before the opening of the Okinawa trough, whereas *F. shibai* most likely showed an ancestral distribution stretching from the central Ryukyus across the Tokara strait to Japanese mainland, whereas *F. churaumi* probably evolved somewhere south of the Tokara strait. Phylogenetic data further indicates that long periods of isolation resulted in heterogeneous genetic structure but subsequent low sea level stands during Pleistocene allowed recent expansion and gene flow between island populations. Comparing these patterns with those of other animals, these tiny wingless mites apparently show better dispersal abilities than partially volant terrestrial organism groups.

## Introduction

The Japanese Islands consist of four major large islands (mainland) and a chain of more than 200 smaller landmasses, the so-called Ryukyus, extending for about 1200 km between the Japanese mainland and Taiwan^1^. All these landmasses are well known for their high species diversity and they are even regarded as global biodiversity hot spots^2^. Reasons for this high biodiversity are (1) the latitudinal range of the island arc stretching across several important biogeographical barriers, as for example the “Tokara gap” between the Holarctic (mainland Japan) and the Oriental region (Ryukyu archipelago), (2) the large number of islands separated by oceanic straits, (3) the humid climate and strong influence of the Asian monsoon and (4) the complex geological history of this region^3^. To put the latter in a nutshell, all Japanese islands once belonged to the Eurasian continent until subduction pulled the Proto-Japanese landmasses eastward, which resulted in the opening of the Sea of Japan approx. 15 Ma ago^4^. Then at 1.5 Ma the Okinawa trough opened leading to the isolation of most southern islands. Some of the islands, however, may have been connected by land bridges during the last glacial periods^5^.

All the above-mentioned factors have shaped the faunas of the Japanese Islands and resulted in a high number of endemic species, some of which are supposed to be relics of immigrants from the Asian continent^6^. The best-known examples of endemic species from Japanese mainland are surely the Japanese macaque, *Macaca fuscata*, and the Japanese giant salamander, *Andrias japonicus*, while the most famous examples from the Ryukyus are the Iriomote-wildcat, *Prionailurus bengalensis iriomotensis*, the short-eared Amami rabbit, *Pentalagus furnessi*^7^ and the recently discovered^8^ and already critically endangered Yambaru long-armed scarab beetle, *Cheirotonus jambar*. In the light of the above, the Japanese landmasses provide a fertile ground for studying vicariance and dispersal of various organisms, especially of non-volant animals with low dispersal abilities.

Oribatid mites are such animals, they are tiny wingless arthropods relying on passive dispersal. Oribatid mites are known to have existed since the early Devonian and presently comprise more than 9000 named species representing 172 families^9^. Most oribatid species are typical inhabitants of various terrestrial habitats, like soil, litter, mosses, trees etc., but a few have also colonized the marine littoral environment. These few comprise only one percent of all Oribatida and most of them belong to the superfamily of Ameronothroidea. Recent studies^10^ suggested this superfamily to represent a paraphyletic assemblage and hypothesized multiple independent evolutionary invasions of the marine littoral environment. The family of Fortuyniidae belongs to the Ameronothroidea and hence represents such an exceptional littoral group that has successfully adapted to the extreme conditions of this fringe habitat. These mites use intertidal algae as substrate and food source^11^ and are able to withstand daily tidal flooding by using an elaborate plastron respiration^12,13^. Presently, there are four genera, *Alismobates*, *Cirellobates*, *Fortuynia* and *Litoribates*, with *Fortuynia*, which currently comprises 15 species, as the most species rich genus.

So far, only a few studies investigated biogeographic patterns of these mites occurring on archipelagos or island groups and their results allow only limited interpretations. Two studies^14,15^, for example, demonstrated morphological divergence between the populations of fortuyniid species from different islands of the Galapagos and the Hawaii archipelago and this divergence was interpreted as a result of limited gene flow between the islands. However, these studies only used morphometric data and genetic evidence was lacking. Another study^16^, on the other hand, demonstrated gene flow between far distant populations of intertidal mites in the Andaman region based on molecular genetic data and hence confirmed long distance dispersal in these tiny organisms. Nevertheless, dispersal mechanisms of these mites are still a controversial issue, but some scientists suggest that long distance transport between islands is mainly achieved by drifting along ocean currents^17,18^. As stated above, the Japanese islands with their geological history and the specific geographic setting with the northwards flowing Kuroshio current offer a reasonable setup to study the distributional and evolutionary patterns of these mites in detail.

Presently there are only three fortuyniid species known from the Japanese islands and none of them is an endemic. The first species was reported from Shikoku and due to its morphological similarity with *Fortuynia elamellata* from New Zealand it was given subspecies rank and named *Fortuynia elamellata shibai*^19^. The second species, namely *Fortuynia rotunda*, was reported from a mangrove forest on Okinawa Island^20^, but was originally discovered in Mozambique^21^. The third species, *Alismobates reticulatus*, was reported from Okinawa and Iriomote Island^20^, but was originally described from Hong Kong^22^. Although these few records remained the only reports of fortuyniid taxa from Japanese shores for the last four decades, these findings indicate vast distribution areas and high dispersal potential of these three species. This is unusual for tiny wingless arthropods, especially against the background of the geographic characteristics and geological history of the Japanese islands.

We now found further populations of fortuyniid taxa on several Japanese islands. Specimens could be identified as the above-mentioned *A. reticulatus* and *F. elamellata shibai*. Additionally, we found specimens of a yet undescribed species that closely resembles the latter. *Alismobates reticulatus* populations were found on the Yaeyama island group near Taiwan and populations of two *Fortuynia* species were found scattered across the Ryukyu archipelago and some parts of Japanese mainland. These new findings finally offered us the opportunity to get a deeper insight into the phylogeography of these mites and to further assess their distributional ranges.

Therefore, the aims of the present study were (1) to confirm and describe the new species, (2) to infer and interpret phylogeographic and distributional patterns of each species in a geological context, (3) to investigate and interpret morphological variation between different islands populations of a single species, and (4) to update biogeographic knowledge about intertidal mites from the Japanese islands.

## Results

### Description of new taxon

Family Fortuyniidae Hammen, 1963.

Genus *Fortuynia* Hammen, 1960.

Type species: *Fortuynia marina* Hammen, 1960.

### Genus diagnosis (updated)

Dark brown mites; interlamellar setae vestigial; notogaster with wide lateral and posterior tectum; lenticulus present; 14–15 pairs of notogastral setae; Van der Hammen’s organ present, epimeral channel *e* absent, channel *q* fully developed, prodorsal channels *ce* and *ci* variable; epimeral setation 3-1-3-2 or 3-1-3-3; five pairs of genital setae; aggenital setae present or absent; three pairs of anal setae and two pairs of anal setae; porose areas on all femora and trochanter III and IV; sexual dimorphism present or absent.

*Fortuynia churaumi* sp. nov. Pfingstl, Shimano & Hiruta.

#### Type material

Holotype: female, Japan, Ryukyus, prefecture Okinawa, Iriomote-jima Island, Ohara on south coast; 16 Mar. 2019; *Bostrychia* and other algae from large rocks on sandy beach. Four paratypes: two males and two females, same location and date as holotype. All deposited at the Collection of Arachnida, Department of Zoology, National Museum of Nature and Science, Tokyo (NMST). Two additional paratypes, from Okinawa-jima, Sosu, Ie no hama; 22 Mar. 2019; *Bostrychia* on rocks, deposited in the collection of the “Senckenberg Museum für Naturkunde Görlitz (SMNG)”.

#### Etymology

The specific epithet consists of the Japanese words “*Chura umi*” which mean *“beautiful sea*” in Okinawa dialect and these words were fused and given as noun in apposition.

#### Diagnosis

Average length 414 μm, mean width 268 μm. Habitus typical for the genus *Fortuynia*. Sensilla short, clavate, bent inwards. Prodorsal canal *ci* absent, *ce* short only reaching bothridium. Notogaster with slightly foveate cuticle. Fourteen pairs of setiform not barbed notogastral setae, *c*_3_ absent. Epimeral setation 3-1-3-2. Aggenital setae present. Conspicuous sexual dimorphism absent.

#### Description of adult

Females (N = 65), length: 394–444 µm (mean 420 µm), width: 252–286 µm (mean 273 µm); males (N = 42), length: 379–425 µm (mean 404 µm), width: 249–274 µm (mean 259 µm).

Integument. Colour dark brown. Cuticle shiny with foveate pattern under dissecting microscope. Cerotegument overall finely granular, larger granules only in acetabular regions. Fine granules forming faint reticulate pattern on all femora.

**Figure 1:**
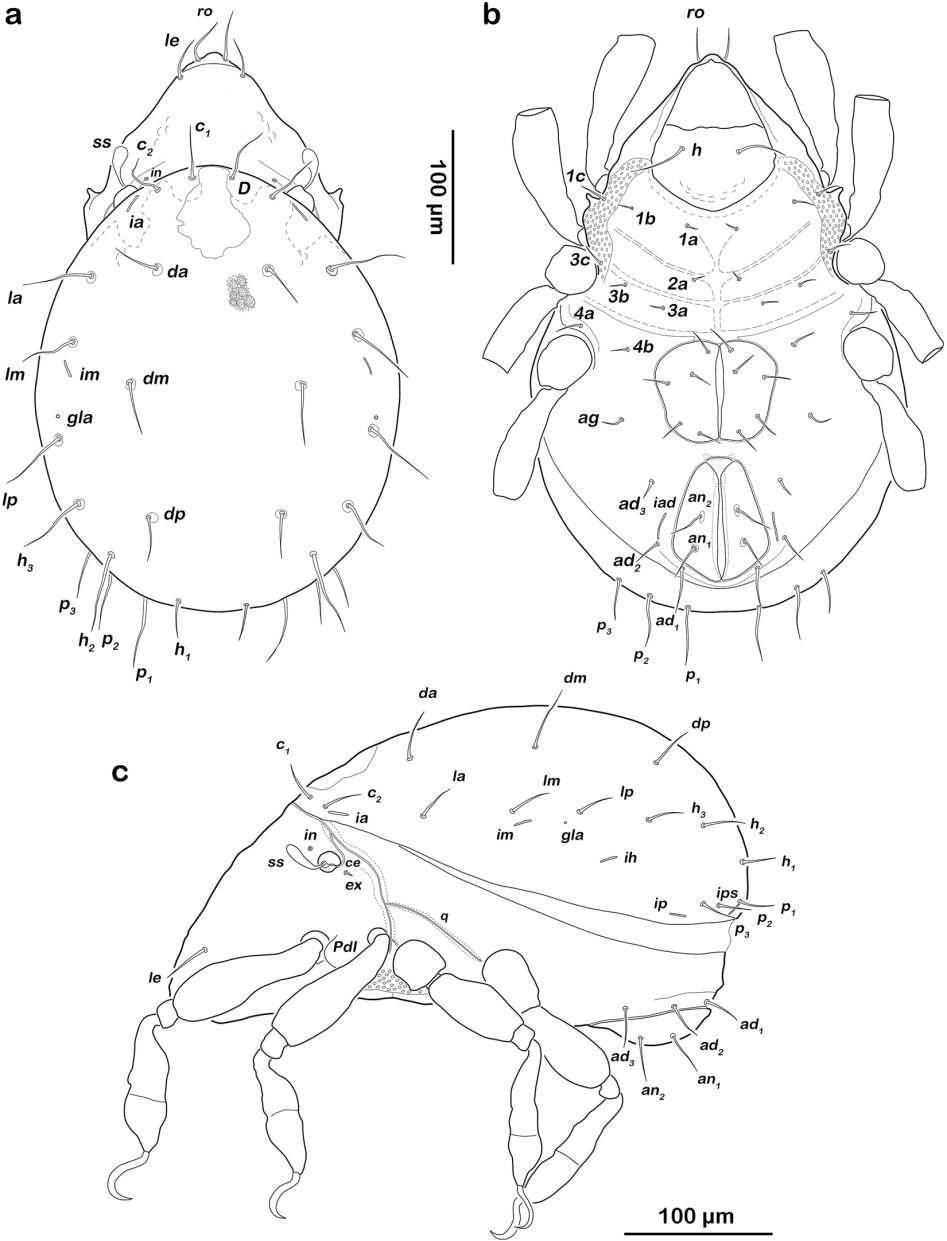
*Fortuynia churaumi* n. sp., adult. (**a**) Dorsal view, legs not drawn. (**b**) Ventral view, gnathosoma and distal leg segments omitted. (**c**) Lateral view, legs drawn simplified.

Prodorsum (Fig. 1a,c). Cuticle dark brown laterally, median interlamellar area lighter coloured. Rostrum triangular in dorsal view, slightly projecting anteroventrally in lateral view. Rostrum demarcated from remainder of prodorsum by faint transverse ridge. Rostral seta *ro* simple, long (approx. 60 µm), lamellar seta *le* thin, shorter (approx. 35 µm). Interlamellar seta *in* vestigial, exobothridial seta *ex* minute and fine. Bothridium small cup, orifice narrow. Sensillum short, smooth, clavate with rounded head bent inwards. Prodorsal channel *ci* absent, *ce* short only reaching bothridium.

**Figure 2:**
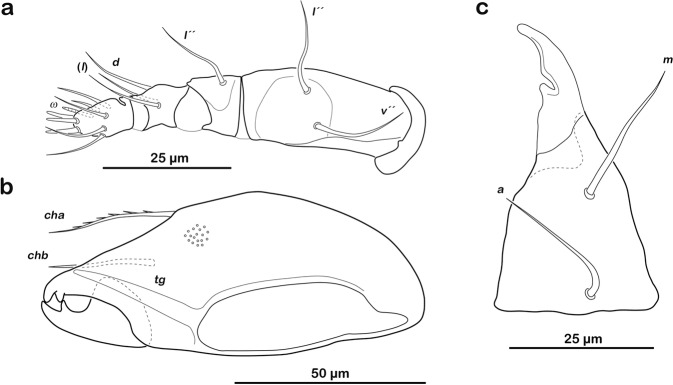
*Fortuynia churaumi* n. sp., adult, mouthparts. (**a**) Left pedipalp, antiaxial view. (**b**) Right chelicera, paraxial view (small circles indicating granular surface pattern). (**c**) Left rutellum, ventral view.

Gnathosoma. Palp typical for family, solenidion *ω* associated (Fig. 2a). Chelicera chelate, with two distinct teeth on each digit. Träghard´s organ *tg* long blade-like lamella with blunt tip. Setae *cha*, *chb* approximately the same length (approx. 18 µm), both dorsally slightly pectinate (Fig. 2b). Gena well sclerotized, distal part of rutellum developed as thin triangular slightly curved inward membrane (Fig. 2c) with longitudinal incision. Setae *a* and *m* long (approx. 25 µm), smooth. Subcapitular seta *h* simple and long (approx. 30 µm).

Gastronotic region (Fig. 1a,c). Notogaster oval in dorsal view. Dorsosejugal suture distinct. Well-developed dorsophragma *D*. Lenticulus elongated with highly irregular borders. Fourteen pairs of thin, simple notogastral setae (length 15–50 µm), *c*_*1–2*_, *da*, *dm*, *dp*, *la*, *lm*, *lp*, *h*_*1–*3_, *p*_*1–3*_; *c*_*3*_ absent. Inconspicuous lighter areas associated with bases of notogastral setae. Five pairs of notogastral lyrifissures present; *ia* next to anterior border of notogaster and seta *c*_*2*_, *im* posterior of seta *lm*, *ih* laterally, *ip* laterad and anterior of seta *p*_3_ and ips next to *p*_2_. Orifice of opisthonotal gland *gla* laterally and between seta *lm* and *lp*.

Lateral aspect (Fig. 1c). Pedotectum I present, rounded but small. Lateral cuticular canals of van der Hammen’s organ typical for genus.

Ventral region of idiosoma (Fig. 1b,c). Epimeral setation 3-1-3-2, all setae simple and short (15–18 µm), *1c*, *3c* and *4a* very close to respective acetabulum. Genital setae setiform, normal length; genital plates rectangular with rounded lateral corners. One pair of aggenital setae present. Anal valves slightly triangular, two pairs of normal anal setae *an*_*1-2*_ (approx. 28 µm). Small ellipsoid or circular lighter cuticular areas at bases of anal setae. Preanal organ triangular. Three pairs of simple, adanal setae *ad*_*1-3*_ (approx. 25 µm). Lyrifissure *iad* slightly oblique, flanking anal orifice.

**Figure 3:**
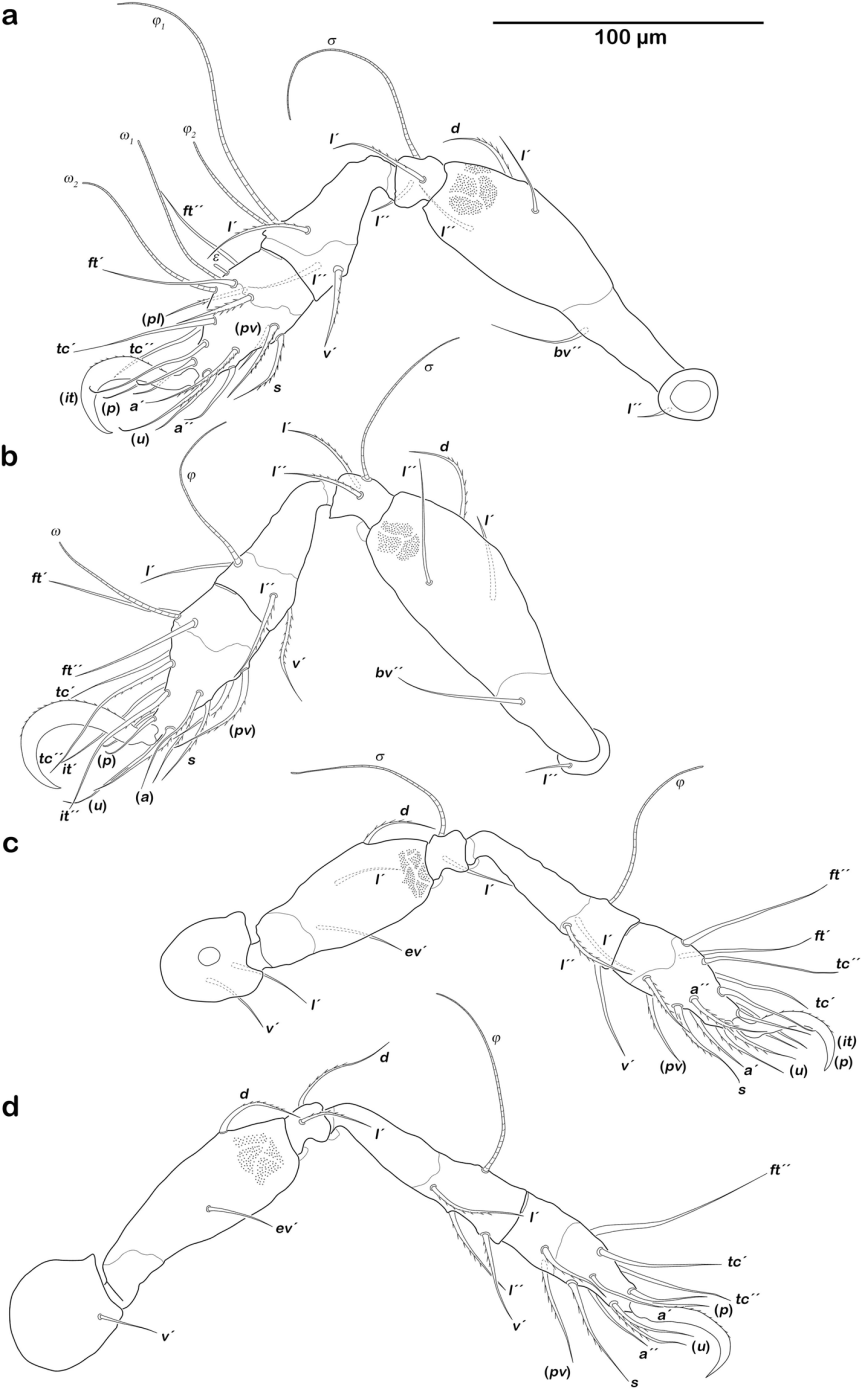
*Fortuynia churaumi* n. sp., adult legs (cerotegumental surface structure indicated on femora; porous areas not shown). (**a**) Right leg I, paraxial view. (**b**) Left leg II, antiaxial view. (**c**) Right leg III, paraxial view. (**d**) Left leg IV, antiaxial view.

Legs (Fig. 3). Long hook-like claws, dorsally faintly serrated. Porose areas typical for the genus. Most dorsal, lateral and ventral setae barbed. Setation and solenidia: Leg I (1-4-2-3-18) (1-2-2), leg II (1-4-2-3-15) (1-1-1), leg III (2-3-1-3-15) (1-1-0), leg IV (1-2-2-3-12) (0-1-0).

#### Distribution

**Figure 4:**
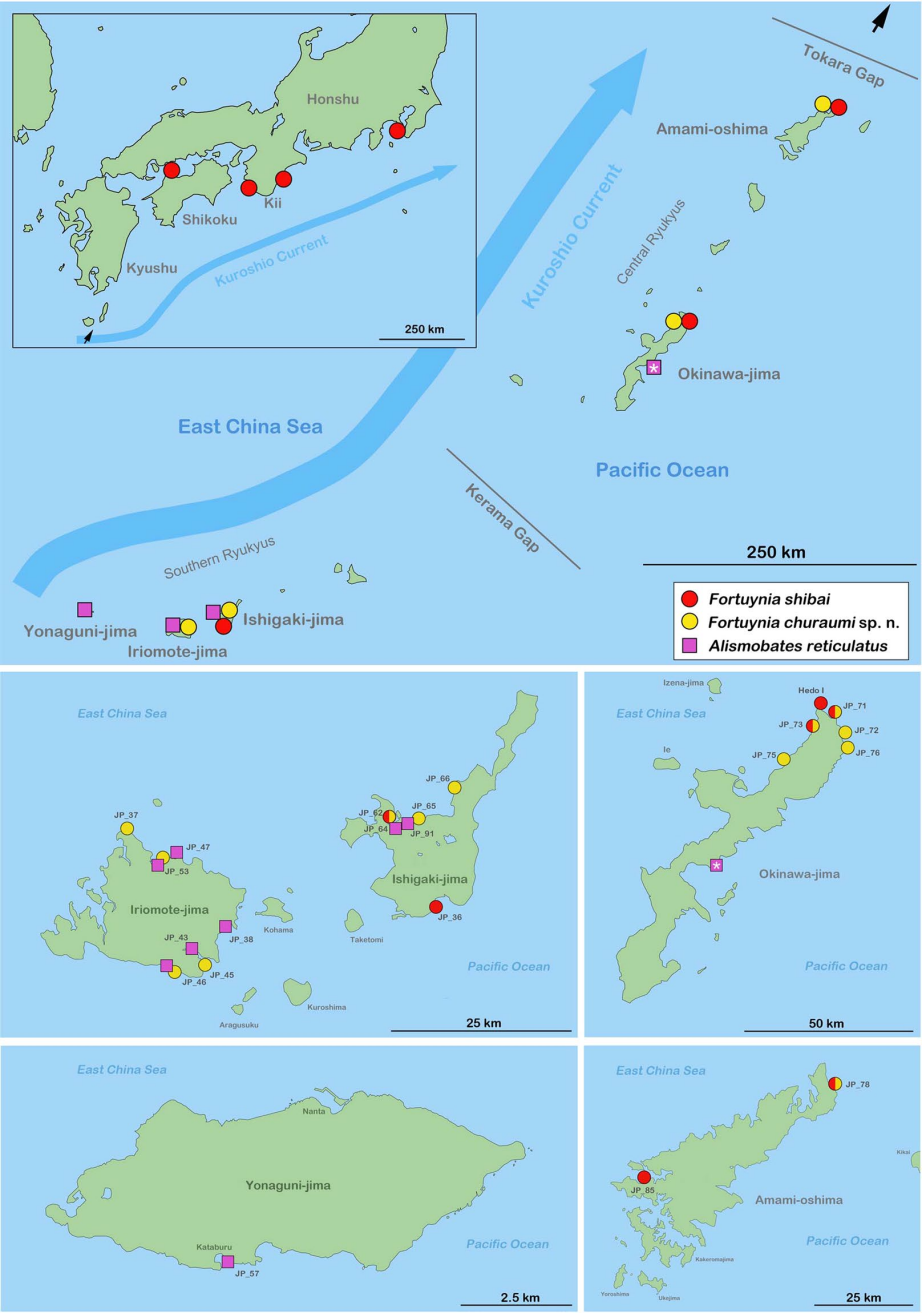
Map showing distribution of fortuyniid species on the Japanese Ryukyus and Japanese mainland. Large map in upper half presenting distribution overview. Insert showing records on Japanese mainland; black arrow pointing to geographic position of area shown in small insert. Occurrence marked with an asterisk represents an earlier published record^20^. Lower four maps showing detailed records of fortuyniid species on five different Ryukyu Islands. Codes refer to sample locations listed in the Material and Methods section. Circles with two colors represent syntopically occurrences. This map was created with the vector graphics editor Inkscape 0.92. (https://inkscape.org).

*Fortuynia churaumi* occurs from the southern Ryukyus (Iriomote-jima, Ishigaki-jima) to the central Ryukyus (Okinawa-jima, Amami-ohshima) (Fig. 4). This species was not found in the northern Ryukyu part north of the Tokara strait yet.

### Remarks

**Figure 5:**
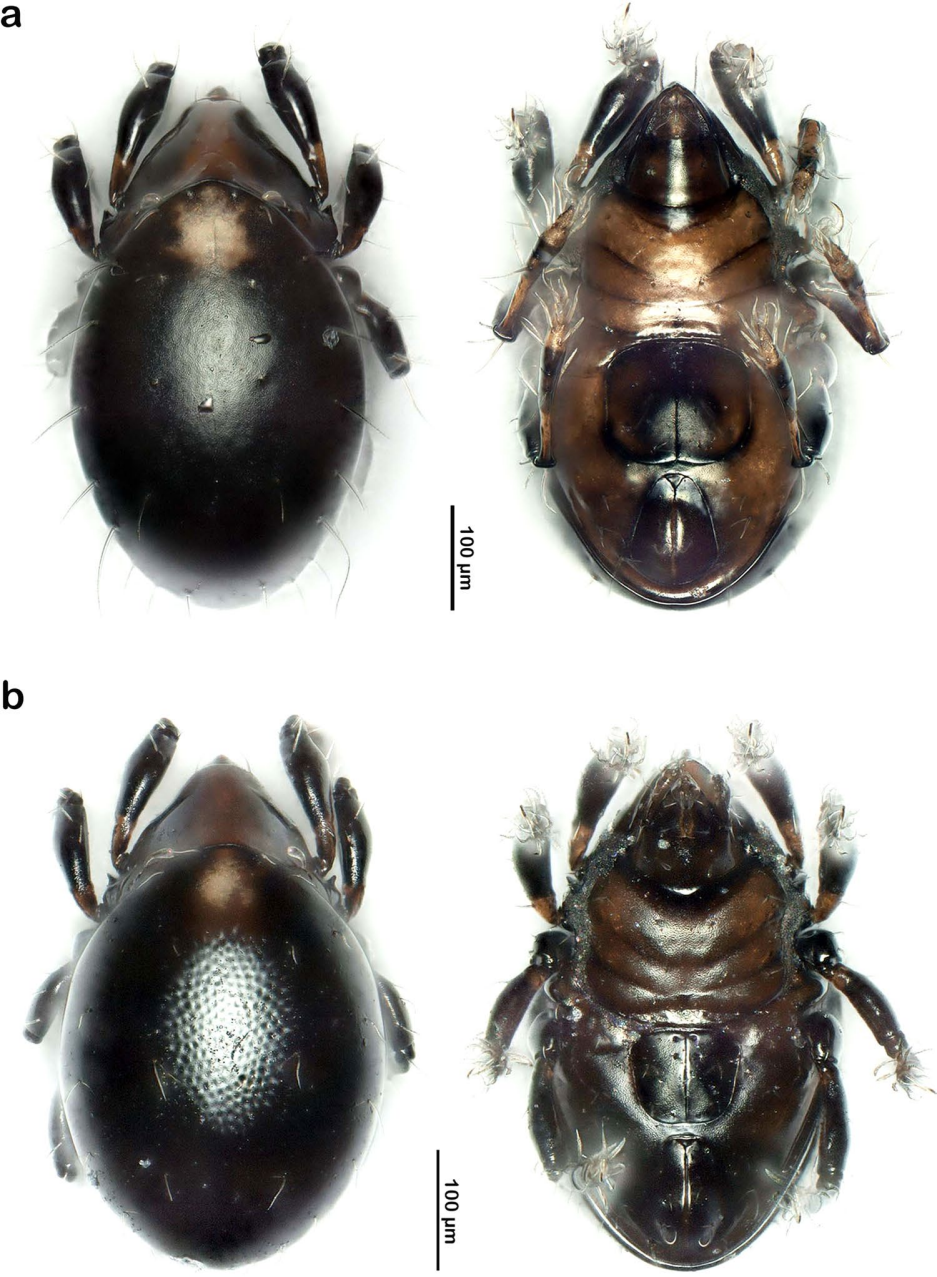
Photographic comparison of found *Fortuynia* species (stacked stereomicroscopic images); left – dorsal view, right – ventral view. (**a**) *F. shibai*. (**b**) *F. churaumi* sp. n.

*Fortuynia elamellata shibai* (Fig. 5a). When Aoki^19^ described this subspecies from Japan, he mentioned differences in the relative length of notogastral setae, position of aggenital seta and the degree of development of pedotecta I and the spur on trochanter III to the nominate species *F. elamellata* from New Zealand, but he considered these divergences only as geographic variation. Given the enormous distance between the occurrence of the subspecies and the nominate species (more than 8.000 km), we think that these differences represent interspecific divergence and not just geographic variants of a single species. Therefore, we promote *F. elamellata shibai* from subspecies to species rank and will refer to it from now on as *Fortuynia shibai*.

#### Genetic data

**Figure 6:**
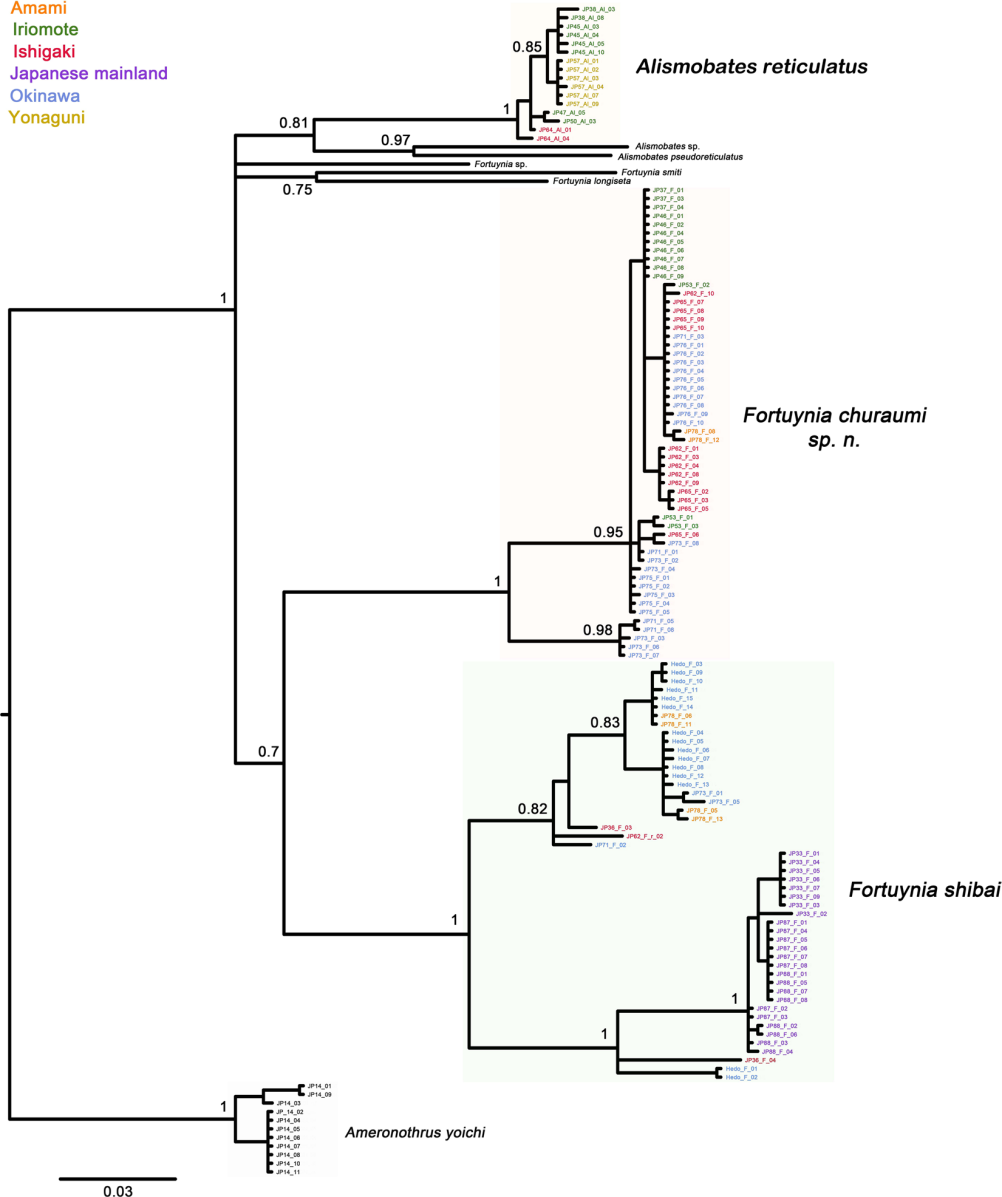
Bayesian inference tree based on the COI sequence data. Numbers at nodes represent Bayesian posterior probabilities (PPs); only PPs >0.7 are shown. Colors of sample codes refer to the Island where the sample was taken; detailed legend shown in the upper left corner.

In both the BI and IQ tree of the COI data, *Fortuynia churaumi* and *F. shibai* represent two distinct clusters (Fig. 6, Supplementary Figure 1), indicating that they indeed represent two distinct species. *Fortuynia churaumi* is quite homogenous, only specimens from western Okinawa-jima form a small but distinct clade. In *F. shibai*, on the other hand, there is a fairly distinct substructure with two large clades, with one of these clades consisting mainly of specimens from the Japanese mainland. *Alismobates reticulatus* forms a distinct clade with no conspicuous substructure. Maximum intraspecific distances in the COI are 14.3, 9 and 2% for *Fortuynia shibai*, *F. churaumi* and *A. reticulatus*, respectively. Mean net distances between species range from 11.9 to 13.5% in *Fortuynia*, with a net divergence of 12% between *F. churaumi* and *F. shibai* (Table 1).

**Table 1:** Estimates of Net Divergence (based on uncorrected p-distances) between fortuyniid species from the Indo-Pacific and Pacific region.

	*F. shibai*	*F. churaumi*	*Fortuynia sp*.	*F. smiti*	*F. longiseta*	*A. reticulatus*	*Alismobates sp*.
*F. shibai*							
*F. churaumi*	0,120						
*Fortuynia* sp.	0,135	0,155					
*F. smiti*	0,119	0,145	0,158				
*F. longiseta*	0,125	0,153	0,151	0,153			
*A. reticulatus*	0,126	0,149	0,169	0,164	0,152		
*Alismobates* sp.	0,149	0,160	0,162	0,151	0,181	0,133	
*A. pseudoreticulatus*	0,128	0,161	0,139	0,155	0,160	0,152	0,132

**Figure 7:**
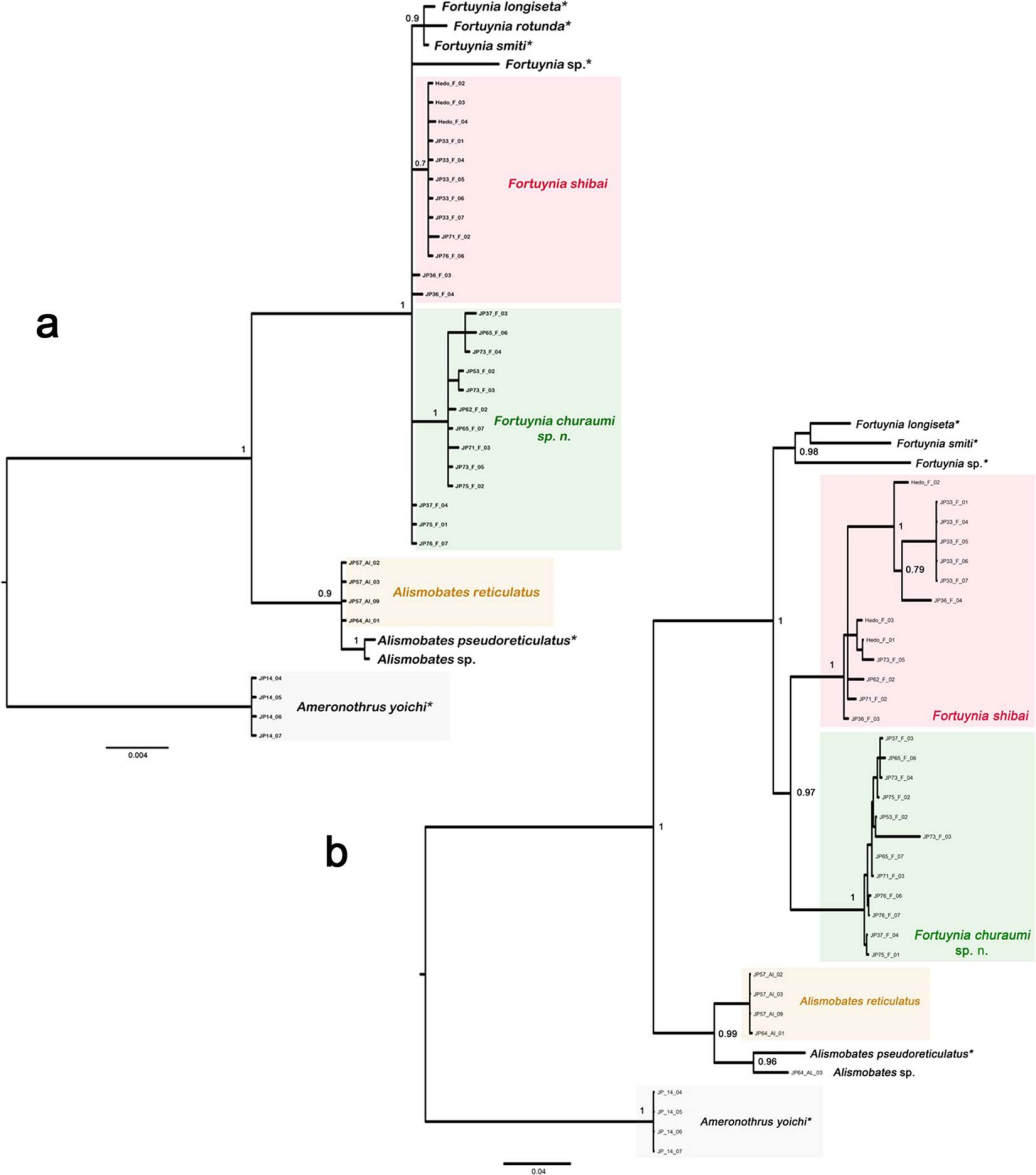
Bayesian inference trees (**a**) based on the 18S rRNA gene of nine fortuyniid taxa and an ameronothroid outgroup. (**b**) based on concatenated data (COI and 18S rRNA sequences) of fortuyniid species and an ameronothrid outgroup. Clades representing Japanese intertidal species are highlighted in colors. *Sequences not generated in this study. Numbers at nodes represent Bayesian posterior probabilities (PPs); only PPs >0.7 are shown.

The BI and IQ trees based on 18S rRNA do not fully resolve *F. churaumi* and *F. shibai* (Fig. 7a, Supplementary Figure 2). In general, although largely consistent with the COI phylogeny, the 18S trees are only poorly resolved. The topologies of both the BI (and IQ) tree of the concatenated data (Fig. 7b) and the multispecies coalescent tree (Supplementary Figure 3) were mostly well resolved, consistent with the single gene trees and a well-supported sister group relationship of *F. churaumi* and *F. shibai*.

**Figure 8:**
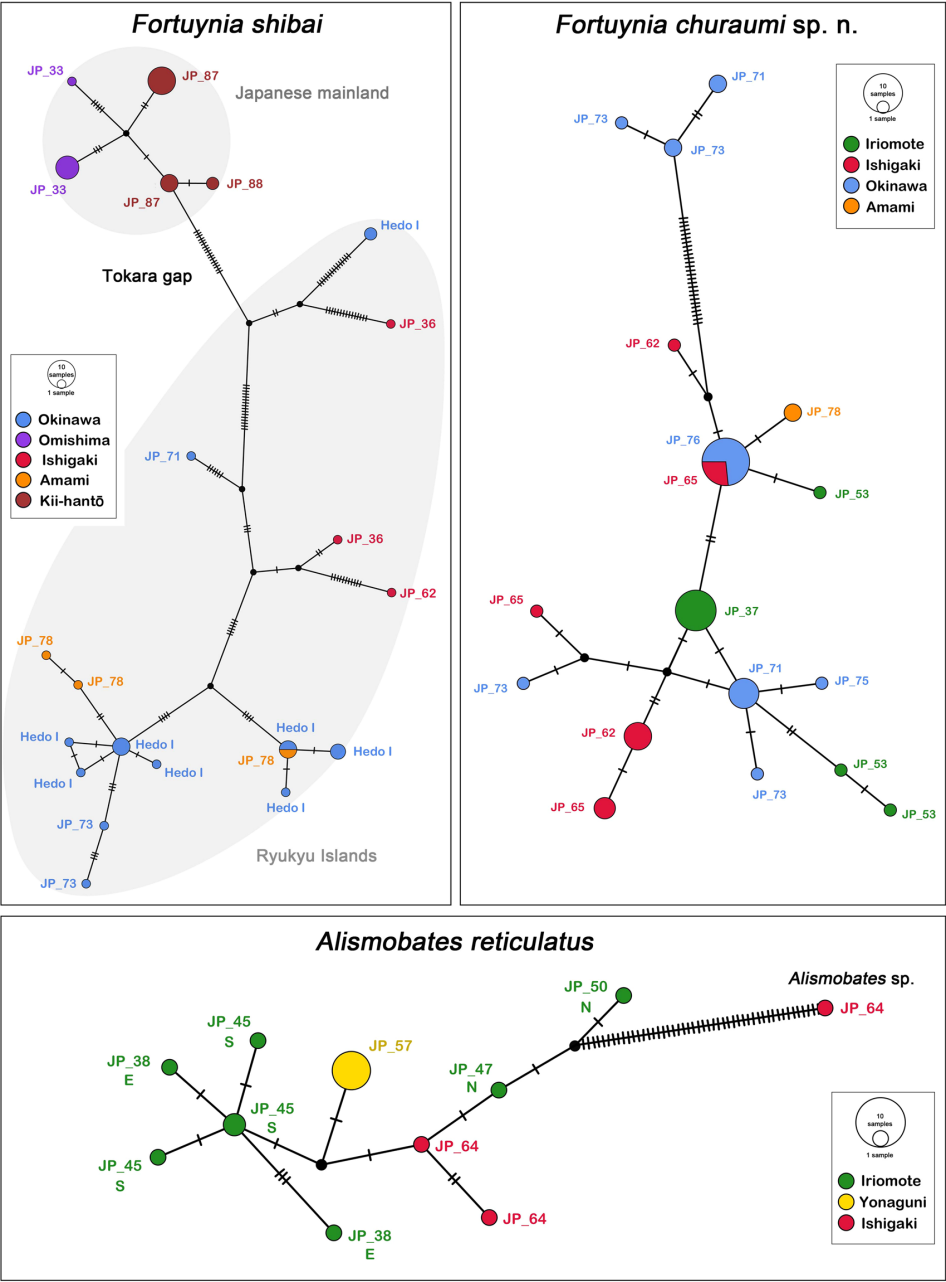
TCS haplotype networks based on COI sequences for three Japanese fortuyniid species. Each circle corresponds to one haplotype and its size is proportional to its frequency, the number of mutations is indicated as hatch marks. Small black circles represent intermediate haplotypes not present in the dataset. Codes refer to sample locations as given in the material and method section; different islands are given in different colors and letters E, N, S (East, North, South) refer to geographic position of location on Iriomote-jima.

TCS analysis resulted in a network of 21 haplotypes for *F. shibai* (Fig. 8), most of them being separated by a high number of mutations. The haplotypes from Japanese mainland form a distinct cluster, but the haplotypes from the Ryukyus show no specific geographic pattern, except for Amami-ohshima, which is closely related to Okinawa-jima, with one shared haplotype between these two islands. The TCS network for *F. churaumi* shows only few mutations between most of the 17 haplotypes (Fig. 8). Only three haplotypes from northwestern Okinawa are distinctly separated from the others, that show no clear phylogeographic pattern. One haplotype is shared between several individuals from Ishigaki-jima and northeastern Okinawa-jima. The *A. reticulatus* network comprises ten haplotypes, which are fairly closely related to each other (Fig. 8). Again, there is no obvious geographic pattern, the northern Iriomote-jima populations are more closely related to the Ishigaki-jima populations and the Yonaguni-jima population lies in between. Only the eastern and southern population from Iriomote-jima form a distinct geographic cluster. However, sample size for this species is certainly too small to allow for any definitive conclusions.

The BPEC (Bayesian Phylogeographic and Ecological Clustering) analysis resulted in the highest support of a two-cluster model of *F. shibai*, one cluster with the most likely ancestral populations on the central Ryukyus and the other cluster on Japanese mainland (Supplementary Figure 4). In *F. churaumi*, this analysis resulted in two clusters with high overlap clusters in the southern Ryukyus with no support for clustering or any ancestral population.

#### Morphometric data

**Figure 9:**
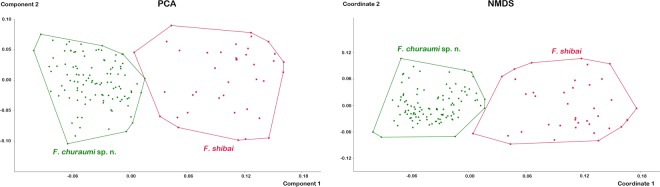
Graph showing results of Principle Component Analysis (PCA) and Non-metric Multidimensional Scaling (NMDS) performed on size-corrected morphometric data of the two Japanese *Fortuynia* species.

Species delimitation/*Fortuynia*. Univariate data (Table 2) shows that overall size differs conspicuously between the two species with the majority of variables being significantly larger in *F. shibai*. In order to reduce this size variation, which may be a result of ecological factors, further species delimitation analyses only used size-corrected data. PCA based on size-corrected data results in a clear separation of both species (Fig. 9) with the first three components accounting for 80.87% of total variation (PC1 52.57%, PC2 20.42% and PC3 7.88%). The variables with the highest loadings on PC1 are genital length *gl* and genital width *gw*, on PC2 lenticulus length *ll* and notogastral width *nw*_*da*_ and on PC3 it was also *nw*_*da*_ and the distance between camerostome and genital orifice *dcg* (Supplementary Table 1).

**Table 2:** Univariate statistics for the two investigated Japanese *Fortuynia* species.

variable	*F. churaumi* sp. n.	*F. shibai*
*bl*	379–444 (414 ± 13.34)	469–525 (504 ± 14.34)
*dPtI*	175–197 (187 ± 4.54)	197–222 (213 ± 5.25)
*db*	99–117 (109 ± 3.80)	99–123 (115 ± 4.87)
*ll*	62–93 (80 ± 6.94)	77–117 (99 ± 12.53)
*nw*_*da*_	200–259 (231 ± 13.37)	234–305 (263 ± 18.23)
*nw*_*dm*_	249–286 (268 ± 9.61)	292–344 (316 ± 12.52)
*nw*_*dp*_	182–252 (213 ± 13.51)	210–292 (263 ± 16.62)
*cl*	114–132 (120 ± 3.44)	120–150 (138 ± 5.56)
*cw*	89–99 (93 ± 2.40)	95–111 (106 ± 3.18)
*dcg*	83–95 (89 ± 3.02)	92–120 (109 ± 5.08)
*dac3*	148–182 (162 ± 6.97)	163–197 (184 ± 5.81)
*gl*	65–86 (75 ± 4.53)	83–123 (111 ± 9.89)
*gw*	86–108 (96 ± 6.02)	111–142 (131 ± 9.23)
*al*	86–102 (93 ± 3.91)	92–114 (103 ± 5.87)
*aw*	68–82 (74 ± 3.27)	80–92 (86 ± 3.63)

Minimum–maximum (mean ± standard deviation) of each measured variable given in µm.

NMDS on size-corrected data shows a distinct morphometric distinction between the two species, with a stress of 0.1553 (Fig. 9). Each species forms a separate cluster without any overlap. PERMANOVA with 10000 permutations reveals highly significant (p < 0.001) differences between the two species.

**Figure 10:**
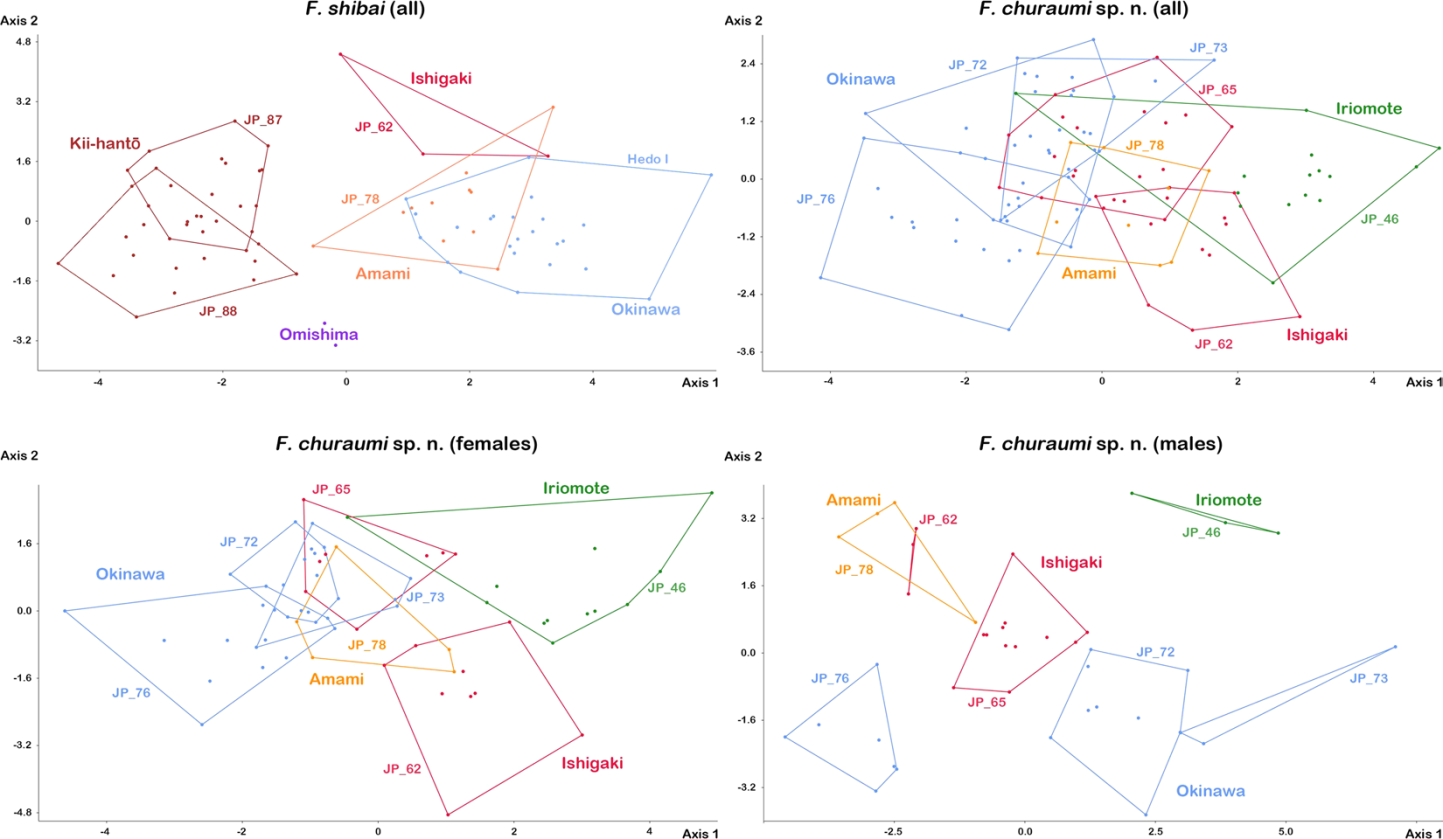
Intraspecific variation of four *Fortuynia shibai* and seven *F. churaumi* sp. n. populations as indicated by a Linear Discriminant Analysis (LDA) performed on raw data. Upper row showing results including all individuals, lower row exhibiting results with *F. churaumi* females and males separated. Populations of the same island are given in the same color. Codes refer to sample locations as given in the material and method section.

Intraspecific variation/fortuynia. LDA on raw data of *F. shibai* results in a clear clustering of the populations, whereas small overlaps are present between Amami-oshima, Ishigaki-jima and Okinawa-jima, as well as between the populations from Kii-hantō (Fig. 10). The two individuals from Omishima and the Kii-hantō populations are clearly separated from all the other populations. LDA can correctly classify 85.9% of the specimens and 60.3% after jackknife validation. MANOVA reveals significant differences between Okinawa-jima and the Kii-hantō populations (p < 0.05). The variables responsible for the displacement of the clusters are *ll* and *dac3*. The Kruskal-Wallis test indicates significant differences in half of the variables between all populations whereas Mann-Whitney U test shows most divergence between the populations from Okinawa and Kii-hantō (Supplementary Table 2).

LDA on raw data of all *F. churaumi* results in a slight displacement of the populations but all show large overlapping areas, whereas the population from Ishigaki-jima (JP_62) does not overlap with the populations from Okinawa-jima (Fig. 10). The highest loading is shown by variable *ll* and 72.64% of the specimens can be correctly classified, only 43.4% jackknifed. Kruskal-Wallis detects significant differences (p < 0.01) in all variables between all populations, except for variables *nw*_*dp*_ and *gl* (Supplementary Table 3). MANOVA reveals significant differences (p < 0.05) between the populations from Iriomote-jima (JP_46) and Okinawa-jima (JP_72, JP_76).

LDA on raw data of only female *F. churaumi* shows a clearer separation of populations. Okinawa-jima, Amami-oshima and one population from Ishigaki-jima (JP_65) still largely overlap but the second population from Ishigaki-jima (JP_62) and from Iriomote-jima form more or less distinct clusters. The variables *ll* and *dac3* show the highest loadings and 76.92% of the specimens can be correctly classified (33.85 Jackknifed).

LDA on raw data of only male *F. churaumi* results in a clear separation of all populations, only the population from Amami-oshima overlaps with the second population from Ishigaki-jima (JP_62) (Fig. 10). Variables *ll*, *gl*, *al* and *aw* show highest loadings and 95.24% can be correctly classified (only 33.33 Jackknifed).

**Figure 11:**
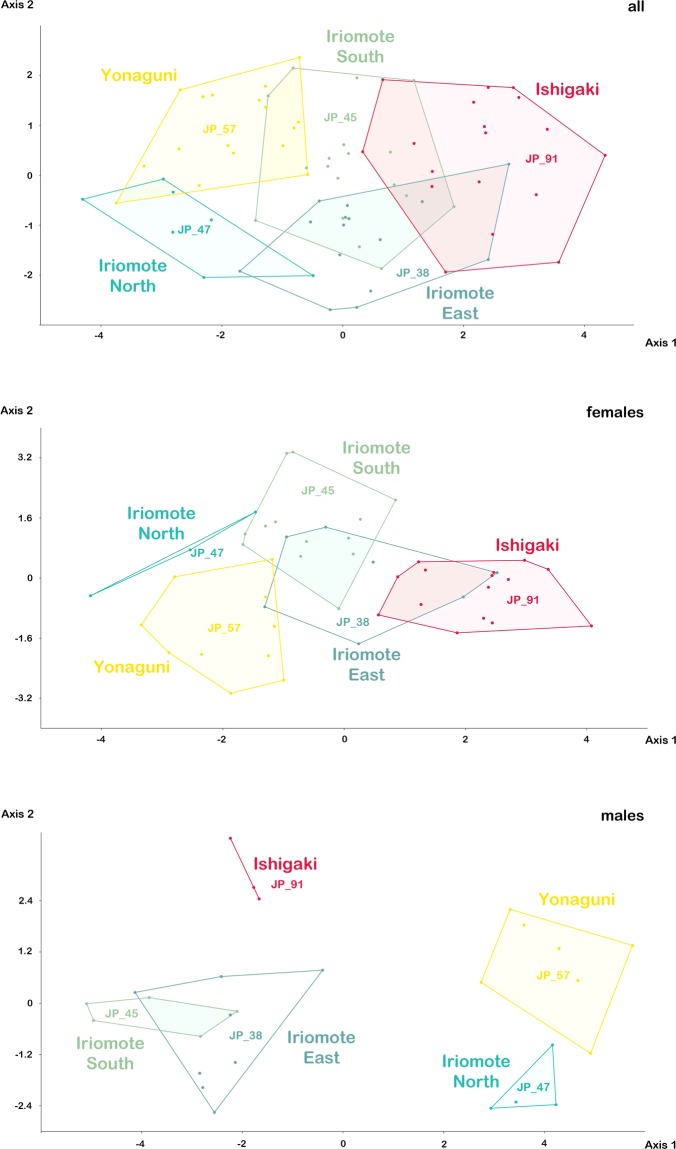
Graph showing results of Linear Discriminant Analysis (LDA) performed on raw data of four *Alismobates reticulatus* populations from two different islands (Iriomote-jima North = JP_47, East = JP_38, South = JP_45; Yonaguni-jima JP_57).

Intraspecific variation/*Alismobates*. LDA on raw data of all *Alismobates reticulatus* specimens yields a more or less distinct clustering with large overlaps between the southeastern populations from Iriomote-jima and the population from Ishigaki-jima and small overlaps between the populations from northern Iriomote-jima and Yonaguni-jima (Fig. 11). Highest loadings are shown by variables *db*, *ll*, *gl* and *gw* and 85.3% of the specimens can be correctly classified (61.3% Jackknifed). Kruskal-Wallis tests find significant differences in nearly all variables, except for *cl* and *dcg*, between all populations (Supplementary Table 4). MANOVA reveals significant differences (p < 0.05) between the Ishigaki-jima (JP_91) and the Yonaguni-jima (JP_57) population.

LDA on raw data of only female *A. reticulatus* shows the same pattern as with all individuals but with less overlapping areas (Fig. 11). Variables with highest loadings are *db*, *ll* and *nw*_*da*_ and 83.3% can be correctly classified (43.8% Jackknifed).

LDA on raw data of only male *A. reticulatus* results in clearly separated clusters, only the population from South and East Iriomote-jima are overlapping (Fig. 11). Only the variable *ll* shows high loadings and 100% of the individuals can be correctly classified (29.6% Jackknifed).

## Discussion

The genus *Fortuynia* is known to show a homogenous morphology, probably as a result of limited habitat preferences^23^, complicating morphological distinction. At first glance, *Fortuynia churaumi* sp. n. and *F. shibai* also look very similar. Nevertheless, both species can be readily distinguished by certain morphological characteristics, i.e. the notogastral setae of *F. shibai* are strikingly thicker and distally weakly barbed (vs. thin and smooth in *F. churaumi*) and the body size as well as the size of the genital orifice are conspicuously larger in this species. The latter differences are nicely reflected in the morphometric data. Additionally, *F. churaumi* shows a foveate notogastral cuticle, a characteristic that is lacking in *F. shibai* (best seen in Fig. 5). High interspecific COI sequence divergence of 12% between the two species also confirms their distinctness. The 18S rRNA data do not resolve the two species as reciprocally monophyletic clusters, but this is most likely the result of the marker’s low mutation rate combined with a recent divergence of the two species, so that insufficient time has passed for complete sorting of lineages (=incomplete lineage sorting^24^).

There are two further species known from East Asian coasts, namely *Fortuynia sinensis* from a Chinese island close to Hong Kong^22^ and *Fortuynia taiwanica* from Taiwan^25^. Both can be easily distinguished from *F. churaumi* and *F. shibai. Fortuynia taiwanica* shows only vestigial posterior genital setae, these are normally developed in both Japanese species and the former exhibits an unusual epimeral setation of 3-1-3-3 instead of 3-1-3-2. *Fortuynia sinensis* is on average 70 µm larger than *F. churaumi*, it shows distally setose notogastral setae and it lacks the foveate notogastral cuticle shown in the latter.

In light of their geographic proximity, a close phylogenetic relationship between all these species could be considered. However, due to the above-mentioned morphological homogeneity of the *Fortuynia*, it is very difficult to infer relationships with morphological data alone. Molecular genetic data, on the other hand, would probably solve this problem, but unfortunately such data are lacking for *F. sinensis* and *F. taiwanica*. So, a clear assessment of the relationships between East Asian *Fortuynia* species must wait until comprehensive molecular genetic data are available.

**Figure 12:**
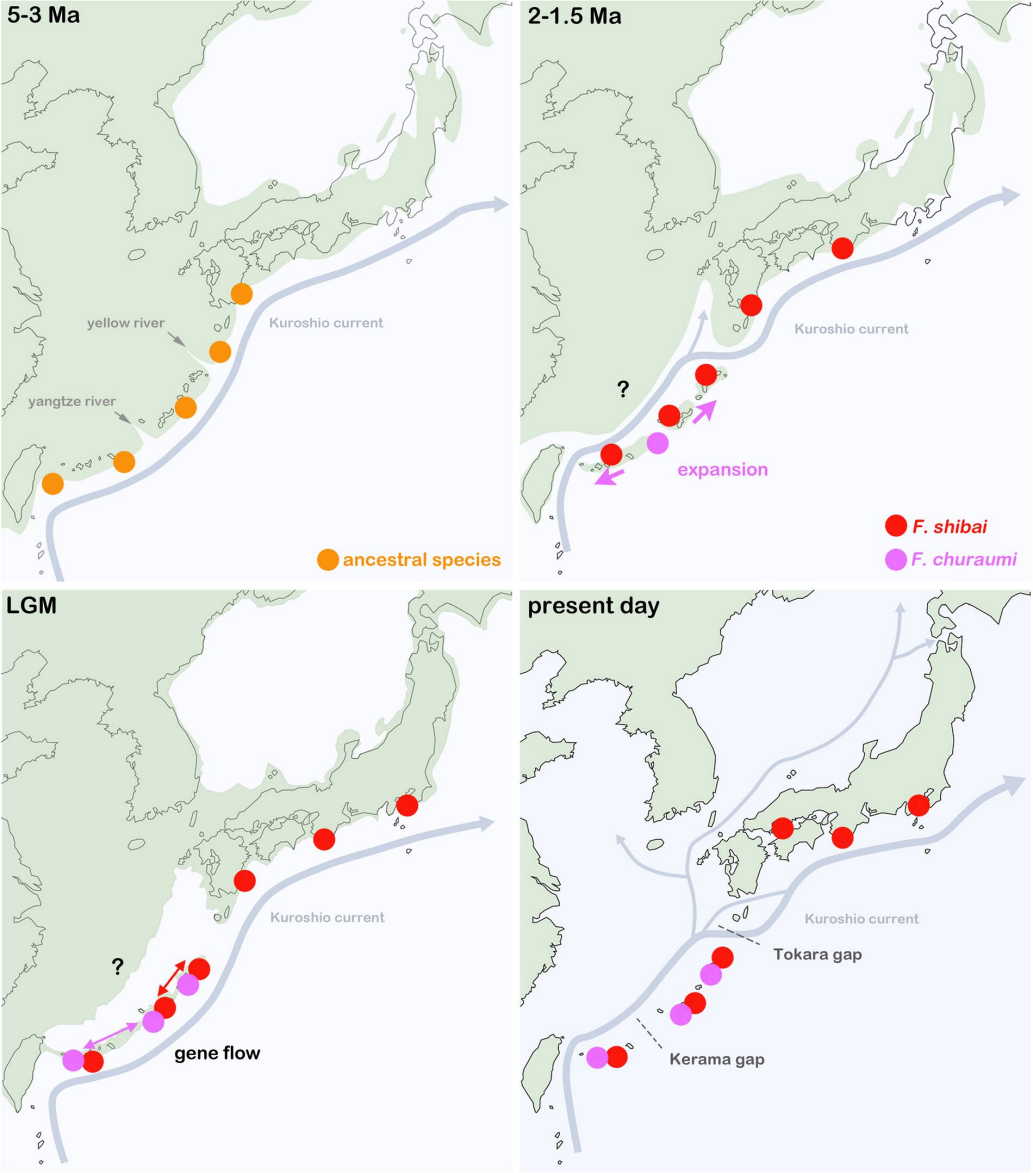
Possible evolutionary scenario inferred from present data and projected on paleogeographic maps: **5–3 Ma** – a common ancestor is distributed along the Asian coastline; close to the end of this period it splits into two distinct species; **2–1.5 Ma** – due to low sea level stands super-islands are formed allowing *F. churaumi* to expand its distribution all over the southern and central Ryukyus; **LGM** – after several periods of isolation most of the Ryukyu islands form a large peninsula resulting in effective gene flow between formerly separated populations; **present day** – populations are again isolated by vast stretches of open ocean. Green shaded areas represent former landmasses; LGM = last glacial maximum approx. 26,500 years ago; ? = former distribution in this area not inferable based on present data. Paleogeographic maps were reconstructed based on data provided by^5,29^. The maps were created with the vector graphics editor Inkscape 0.92. (https://inkscape.org).

Based on present morphological and molecular genetic data, *F. shibai* and *F. churaumi* are clearly sister species that are derived from a common Eastern Asian ancestor. Unfortunately, there is no reliable substitution rate available for the COI gene of mites, therefore linking the speciation of these two taxa with a geological event is not unambiguously feasible. However, previous attempts^26,27^ to infer divergence times in oribatid mites used a general arthropod substitution rate of 2.15% per Ma and applying this rate to the present data would result in a speciation event 2.8–3 Ma ago, prior to the formation of the Okinawa trough, when the Japanese landmasses were still part of the Asian coastline^4^. The present distribution ranges, with *F. shibai* occurring from the Ryukyus to Kanagawa on Honshu and *F. churaumi* being restricted to the southern and central Ryukyus, indicate that *F. shibai* most likely showed an ancestral distribution stretching from the central Ryukyus across the Tokara strait to Japanese mainland, whereas *F. churaumi* probably evolved somewhere south of the Tokara strait (Fig. 12).

The topology of the phylogenetic trees and the haplotype network suggest that most *F. shibai* populations were separated for a long period in their evolutionary history. The Japanese mainland populations are genetically distinct from those of the southern and central Ryukyus which indicates that the opening of the Tokara gap in the early Pleistocene^6^ might have separated these population groups and has acted since then as effective geographic barrier. The populations from the Ryukyus show considerable genetic structure and divergence, but without a clear geographic pattern which might be the result of a complex geological history. Most islands of the Ryukyus are considered to have been isolated from each other for long periods^5^ but in the late Pleistocene super islands and land bridges emerged, connecting these landmasses again for a short period^28^. This geological sequence would first produce genetically distinct lineages on the islands that then would come into secondary contact, a scenario consistent with the pattern observed in the *F. shibai* data. Moreover, the close and partly shared haplotypes between the Amami-ohshima and the Okinawa-jima populations fit the assumption that these central Ryukyu islands have been connected during low sea level stands in the late Pleistocene^28,29^. Another explanation for this close relationship could be gene flow caused by recent dispersal. Long distance transport of intertidal mites is suggested to happen mainly by passively drifting along ocean currents^17,18^ and the northwards flowing Kuroshio current may indeed facilitate dispersal between these neighboring island groups. A similar relationship between populations from Amami-ohshima and Okinawa-jima was shown in the tideland snail *Batillaria multiformis* but in this case the authors^30^ suggested a dispersal in the opposite direction with a southwestward current. However, based on our data we cannot conclude whether former super-islands or oceanic dispersal is responsible for the close relationship of *F. shibai* populations of the central Ryukyus.

Apart from a close relationship between Amami-ohshima and Okinawa-jima, *F. churaumi* shows a different phylogeographic pattern, with only minor genetic divergence among all islands. The fact that the sequences of this species have not diverged considerably even among remote islands suggests that expansion has occurred fairly recently. The formation of a land bridge connecting Taiwan with the southern and central Ryukyu Arc during the last glacial maximum was postulated^29^, but this paleo-peninsula would have been too recent to explain the observed pattern. However, this pattern could very well be the result of a similar large paleo-peninsula extending from China to the northern Ryukyus that already existed in the early Pleistocene (1.7-1.2 Ma)^1^. So, *F. churaumi* populations may have dispersed across the Ryukyu islands along this large early Pleistocene paleo-peninsula whereas the land bridge in the late Pleistocene again allowed gene flow between populations, resulting in the shared haplotypes between southern and central Ryukyus. Moreover, the present distribution of *F. churaumi* does not extend beyond the Tokara gap and hence corresponds with these suggested early and late Pleistocene landmasses.

Apart from all the closely related island populations, there is a single lineage of two *F. churaumi* populations from northern Okinawa-jima which is genetically distinct. This small clade may either represent a relic of a more ancient population, which would imply that this species already existed on the central Ryukyus before the formation of the early Pleistocene paleo-peninsula, or it may be the result of a recent colonization event from a more isolated area of this region. If *F. churaumi* already existed on the central Ryukyu islands before the Pleistocene then the lack of further relic populations could only be explained by their extinction. Although possible, we favor a recent colonization event from a far distant area. The Kuroshio current passes Taiwan, flows through the Okinawa trough and enters the Tokara strait before flowing into the Pacific Ocean^31^. A dispersal along this route from Taiwan or other southern areas to northern Okinawa-jima may be possible. Indeed, garbage of Taiwanese origin is regularly washed ashore in the exact area of our sampling sites in northern Okinawa-jima (unpublished data, personnel communication with local authority). Nevertheless, answering this question would require sequence data of *F. churaumi* populations from supposed southern areas of origin, e.g. Taiwan, but these are presently lacking.

*Alismobates reticulatus* shows a distribution from Hong Kong to the central Ryukyus^20,22^, but the present study only provides molecular genetic data for populations from the southern Ryukyus (Yaeyamas), so conclusions about the evolutionary history can only be made in a geographically limited context. Haplotypes from this insular group show a fairly homogeneous structure. The islands of this group are suggested to have been separated from each other since approx. 1.5 Ma^5^ and thus distinct genetic lineages could be expected. However, as already mentioned above, these landmasses were most likely all part of a large peninsula or super-island during low sea level stands of the Pleistocene^29^. Therefore, the pattern we find makes absolute sense. Moreover, Iriomote-jima and Ishigaki-jima are separated by very shallow coral sea, so they can be treated as a single island in paleogeographic studies^5^. Interestingly, the eastern and southern populations from Iriomote-jima appear to be more closely related to the Yonaguni-jima population than to the northern Iriomote-jima populations. The latter are located at the East China Sea coast and the former are all facing the Pacific Ocean. Considering the paleo-peninsulas during the Pleistocene^29^, these populations would have been on completely different coastlines during these periods which could explain a close relationship between the populations from eastern and southern Iriomote-jima and Yonaguni-jima. However, more data are needed to test this hypothesis. Nevertheless, it demonstrates that the continuous coastline of a small island does not necessarily imply a continuous gene flow and that populations obviously do not migrate long distances on land along a coastline.

Comparing the phylogeographic pattern of all herein studied intertidal mites with other animal taxa, we can find similarities but also some conspicuous differences. The Tokara Strait and the Kerama gap represent effective zoogeographical barriers for several insects^3^, amphibians, reptiles^32^, birds^33^ and mammals^7^. The Tokara gap is also a clear barrier for the mites, as shown by molecular genetic, morphometric and distributional data. The Kerama gap, on the other hand, is not reflected as such in the present data. Apparently, those tiny little wingless mites do have better dispersal abilities than the above mentioned, partially volant terrestrial organism groups. The key for their successful dispersal is unknown but may be based on their suggested ability to drift on water^17^. Even if they are not transported over large distances, rafting from one location on an island to another close location may facilitate dispersal in the long term, especially in the case of geologically alternating coastlines.

Morphometric data demonstrates that there is considerable variation between the different island populations of each species. In *F. shibai* the specimens from Japanese mainland are clearly distinct from the Ryukyu populations, consistent with a long-lasting separation by the Tokara gap. The variation among the populations from the Ryukyus also corresponds to geography and molecular genetic data showing a closer relationship of Okinawa-jima and Amami-ohshima, both islands of the central Ryukyus. In *F. churaumi* and *A. reticulatus* remarkable variation between the populations from different locations is present, which also correlates to a certain extent with the geographic setting and the genetic data. For example, like with the genetic data, populations of *F. churaumi* from Okinawa-jima and Ishigaki-jima cluster together also based on morphometric data. The close relationship of southern and eastern populations of *A. reticulatus* from Iriomote-jima is also reflected by morphometric data.

Interestingly, males of *F. churaumi* and *A. reticulatus* show more morphological divergence than females, hence allowing a better separation of the populations. These species show a weak sexual dimorphism with females being slightly larger and possessing larger genital orifices, which results in the blurred picture when both sexes are analyzed together. Another study found the opposite result, with females of *Alismobates galapagoensis* showing more variation between the islands of the Galapagos archipelago than males^15^. Apparently, there is no general rule and the reason for this gender-related variation remains unknown. The latter study^15^ also revealed a correlation of morphological divergence with geographic distance, i.e. closer populations show less variation than more distant populations. The herein found variation does not reflect such a clear geographic pattern as close populations of a single island show partly more divergence than populations from distant islands. But considering the geological history of the Ryukyus, with islands being separated and connected again, the pattern should clearly differ from populations of a volcanic archipelago, like Galapagos, where islands emerge subsequently and remain more or less isolated after being colonized.

Both Japanese *Fortuynia* species appear to have a strict habitat preference as they were, without exception, found in the intertidal alga *Bostrychia* that grows on rocky substrate in the lower or middle eulittoral zone. The genus *Fortuynia* is known to be mainly restricted to rocky coasts and to show habitat conformity^23^, hence this is not unusual. What is unusual, is the fact that *F. churaumi* and *F. shibai* occurred syntopically at four different locations on three different islands, which means they were found in the exact same small patch of algae (ca. 10 cm^2^). There are several records of syntopically occurring intertidal mites^34,35^, but these were never closely related congeneric species. In the present case, we can only speculate why and how these species share the same microhabitat. Either they feed on different parts of the algae or on other microscopic food within the algae (e.g. coccal algae, bacteria), or they show a vertical zonation and the respective samples were incidentally taken in the area of overlap. Another possibility is that there is no niche separation at all and that both species compete for the same habitat and food resource. Indeed, there are certain aspects in the biogeographic and phylogeographic pattern pointing to such a scenario. *Fortuynia churaumi* has expanded its distribution range all over the southern and central Ryukyus in the early and late Pleistocene and hence may have recently invaded the habitats formerly occupied by *F. shibai*. Additionally, the latter species is the only one that could not be found on Iriomote-jima, which is very unusual because it is present on Ishigaki-jima. Both these islands are part of the Yaeyamas, a group of closely located neighbor islands, which were all part of a larger island during the Miocene and during several periods of the Pleistocene^1,6^. The apparent absence of *F. shibai* on Iriomote-jima could be a result of *F. churaumi* having already replaced this species on this island. If this is the case, then *F. shibai* might also vanish from the southern and central Ryukyus in the near future but may further exist on the northern Ryukyus and Japanese mainland as long as *F. churaumi* does not successfully cross the Tokara gap.

In contrast to the narrow habitat range of the two *Fortuynia* species, *A. reticulatus* shows a wide variety of habitat types. It was found in diverse intertidal algae, in both rocky shore and mangrove forest, whereas rocky shore samples ranged from large cliffs, to small boulder, to man-made concrete structures. Not only the habitat type but also the vertical distribution within each type showed wide ranges. Specimens of *A. reticulatus* could be found from the lower to the upper eulittoral close to the beginning terrestrial vegetation on a rocky shore, and from the floor to the bark of the lower trunk section in a mangrove forest. An earlier study^23^ already indicated that *A. reticulatus* may be a generalistic inhabitant of the littoral area and the present data clearly confirm this.

## Material and Methods

Samples of littoral algae were scraped off rocks, concrete walls and mangrove roots with a knife or a small shovel and then put in Berlese-Tullgren funnels for 12 to 24 hours to extract mites. Afterwards, collected specimens were stored in ethanol (100%) for morphological and molecular genetic investigation. The majority of samples was taken by Shimano, Hiruta and Pfingstl and if not, the name of the collector is provided.

### Sample locations

The Ryukyu arc is geographically and geologically subdivided into the northern, central and southern section by the Tokara strait, Kerama Gap and Yonaguni Strait, respectively^36^. In the central Ryukyus we collected samples from Amami-ohshima and Okinawa-jima and in the southern Ryukyus we took samples from Yonaguni-jima, Iriomote-jima and Ishigaki-jima, these islands are also known as the main Yaeyama islands. For a better understanding of the geographic setting please refer to the figures showing the maps.

Each sample location was given a code; these codes are presented in parentheses and will be used throughout the manuscript to allow easier linking of geographic information. The suffixes ‘‘-jima/-shima’’,"-kawa/-gawa" and “-hantō” are Romanized Japanese meaning ‘‘island’’, “river” and “peninsula” respectively; in the graphs and tables showing results these suffixes are not given due to a shortage of space.

1. Misaki-hantō, pref. Kanagawa: Jyoga-shima : intertidal algae on rock; 35°07′44.3″N 139°37′41.2″E; 13 Nov. 2016, leg. S. Shimano & J. Shimano.
2. Kii-hantō, pref. Wakayama: Shirahama-cho, Nishinmuro-gun (JP_87), Seto Marine Biological Laboratory, Field Science Education and Research Center; 33°41′32″N 135°20′04″E; 18 Jun. 2019. Ugui, Nachi-Katsuura city (JP_88), intertidal algae from rocks; 33°39′19.13″N 135°58′44.83″E; 23 Apr. 2019, leg. K. Akita.
3. Omi-shima, pref. Ehime: (JP_33) black intertidal algae on mussels and barnacles on concrete wall; 34°13′26.60″N 132°59′0.68″E; 17 Sept. 2018.
4. Iriomote-jima, pref. Okinawa: Starsand Beach (JP_37) dark green algae from large rock on sandy beach; 24°26′11.22″N 123°46′36.80″E; 15 Mar. 2019. Goyoshi-gawa estuary  (JP_38) brown filamentous algae on mangrove floor, 24°19′17.43″N 123°54′38.87″E; 16 Mar. 2019. Nakama-gawa (JP_43) bark and brown algae from mangrove roots; 24°17′43.60″N 123°51′49.83″E; 16 Mar. 2019. Ohara (JP_45) diverse algae growing on concrete pier; 24°16′32.38″N 123°52′58.87″E; 16 Mar. 2019. South shore (statue of the teacher) (JP_46) *Bostrychia* and other algae from large rocks; 24°16′0.02″N 123°50′45.71″E; 16 Mar. 2019. Nadara-gawa estuary (JP_47) *Bostrychia* and other algae from boulder; 24°23′52.93″N 123°49′42.72″E; 16 Mar. 2019. Uehara (JP_50) *Bostrychia* and other algae on artificial stone wall; 24°23′49.18″N 123°49′19.59″E; 17 Mar. 2019. Uehara (JP_53) *Bostrychia* from rocky shore; 24°23′53.48″N 123°49′20.21″E, 17 Mar. 2019.
5. Yonaguni-jima, pref. Okinawa: Kataburu Beach (JP_57) *Bostrychia* and smaller plants on small rocks; 24°26′25.78″N 122°58′27.44″E; 18 Mar. 2019.
6. Ishigaki-jima, pref. Okinawa: Ohama Beach (JP_36) *Bostrychia* on rocks on sandy beach; 24°20′40.61″N 124°12′5.58″E; 15 Mar. 2019. Kabira Bay (JP_62) *Bostrychia* on large rocks on sandy beach; 24°27′49.14″N 124° 8′39.09″E; 20 Mar. 2019. Kabira Bay (JP_64) *Bostrychia* and diverse algae from small stones; 24°26′33.63″N 124° 8′29.79″E; 20 Mar. 2019. Yamabare (JP_65) thick cushions of *Bostrychia* on large rocks; 24°26′56.11″N 124°10′46.41″E; 20 Mar. 2019. Fukido-gawa (JP_66) estuary with large mangroves, diverse algae growing on mangrove roots; 24°29′10.48″N 124°13′48.29″E; 20 Mar. 2019. Kabira Bay (JP_91) algae on rocks; 24°26′56.2″N 124°08′47.3″E; 31 Mar. 2019; leg. H. Uchida.
7. Okinawa-jima, pref. Okinawa: Oku (JP_71) *Bostrychia* growing in crevices of rocky shore; 26°50′44.60″N 128°17′22.00″E; 22 Mar. 2019. Sosu (JP_72) *Bostrychia* in large crack on huge single rock; 26°48′30.23″N 128°19′6.00″E; 22 Mar. 2019. Sosu, Ie no hama (JP_76) *Bostrychia* on rocks; 26°47′40.24″N 128°19′7.65″E; 22 Mar. 2019. Uka (JP_73) *Bostrychia* on rocks; 26°49′50.91″N 128°14′43.35″E; 22 Mar. 2019. Cape Okuma (JP_75) large cushion of *Bostrychia* on huge rock; 26°43′47.66″N 128° 9′38.15″E; 22 Mar. 2019. Uzabama, Kunigami Village, Cape Hedo (Hedo I) *Bostrychia* on rocks; 26°51′50.4″N 128°15′56.3″E; 12 Sept. 2018.
8. Amami-oshima, pref. Kagoshima: Cape Ayamaru (JP_78) *Bostrychia* from crevices on rocks (rocky shore); 28°28′23.36″N 129°43′8.03″E; 23 Mar. 2019. Nagara Bay (JP_85) black bushy algae on concrete cay wall; 28°15′21.20″N 129°12′7.56″E; 24 Mar. 2019.

### Molecular genetic analyses

Whole genomic DNA was extracted from 121 ethanol fixed specimens of the family Fortuyniidae following to the modified Chelex protocol^16^. A ~566 bp long fragment of the mitochondrial DNA cytochrome oxidase subunit I (COI) and the complete 18S rRNA (~1715 bp long) locus were amplified^36–40^ (for information on primers see Supplementary Table 5). Post-PCR purification steps included enzymatic ExoSAP-IT (Affymetrix). Cycle sequencing was applied according to^41^ using the BigDye Sequence Terminator v3.1 chemistry (Applied Biosystems). Sequencing products were purified with Sephadex G-50 (GE Healthcare). Capillary sequencing and sequence visualization were conducted on an ABI 3130xl (Applied Biosystems) automatic sequencing device. The sequences have been deposited on GenBank under the accession numbers MN385024 – MN385144 (COI) and MN372404 - MN372433 (18S rRNA) (see Supplementary Table 6).

Nucleotide sequences were aligned in the software MEGA v7.0^42^ using implemented MUSCLE algorithm^43^. Additionally, already published sequences were downloaded from GenBank and added to the alignment (see Supplementary Table 6). Intra- and interspecific genetic distances were calculated based on uncorrected p-distances in MEGA v7.0. Tree searches based on Maximum likelihood (ML), using IQ-TREE v1.5.5^44^, and Bayesian inference (BI), in MrBayes v3.2^45^, were conducted on three different datasets: (i) COI, (ii) 18S and (iii) COI + 18S. The best-fitting data partitioning schemes and models of molecular evolution were determined based on the Bayesian Information Criterion (BIC) under the “greedy” algorithm in PartitionFinder v2.1.1^46^. ML tree search was conducted with 30.000 ultrafast bootstrap replicates^47^ in IQ-tree. Posterior probabilities for BI were obtained from Metropolis-coupled Markov chain Monte Carlo (MCMC) simulations using 2 independent runs, 8 chains, 15 million generations and a sampling frequency of 10 000. The first 25% of trees were discarded as burn-in and posterior trees were summarized as a 50% majority rule consensus tree. Convergence of parameters and stationarity of chains were assessed by split deviation frequencies (<0.01) and effective sample sizes (ESS) in Tracer 1.6^48^ (available at http://beast.bio.ed.ac.uk/Tracer). Both the IQ and BI tree were visualized in FigTree v1.4.2^49^ (available at http://tree.bio.ed.ac.uk/software/figtree).

Additionally, a StarBeast2 - multispecies coalescent analysis^50^ - was conducted in BEAST v.2.4.7^51^. MCMC settings and priors were organized in BEAUti with a population model set on *Analytical Population Size Integration*, gene ploidy according to locus, *YULE*-model and uncorrelated lognormal relaxed clocks for both loci, a chain length of 300 million generations (sampling frequency of 10 000) and best-fitting substitution models based on model-search in PartitionFinder (Table 3). Chain convergence and stationarity were assessed in Tracer 1.6. The MCMC output was summarized (using a relative burn-in of 25%) as a maximum-clade credibility tree in TreeAnnotator v2.5.0^52^ and visualized in FigTree v1.4.2.

**Table 3:** Dataset details, partitioning schemes and best-fitting substitution models obtained from the greedy algorithm in PartitionFinder.

Dataset	Length	N	Partitioning	Model
(i) COI	425 bp	137	No partitioning	GTR + I + G
(ii) 18S	1715 bp	38	No partitioning	K80 + I + G
(iii) COI + 18S	2187 bp	38	(1) 1^st^ COI + 2^nd^ COI + 18S	(1) HKY + I + G
			(2) 3^rd^ COI	(2) HKY + G

Furthermore, subsets of the COI dataset (i) were used for constructing intraspecific haplotype networks and phylogeographic analyses. TCS networks^53^ of (a) *Alismobates reticulatus* (N = 17, 425 bp), (b) *Fortuynia shibai* (N = 49, 439 bp) and (c) *F. churaumi* sp. nov (N = 55, 439 bp) were built in PopArt 1.7^54^. To determine geographical population clusters and ancestral locations, the R-package “bayesian phylogeographic and ecological clustering” (BPEC)^55^ was performed on the datasets (b) and (c) in R 3.1.2^56^. The analyses were conducted on a two-dimensional dataset (longitude, latitude), allowing one migration event and with the strict parsimony option (ds = 0) selected. 1000 samples were saved from two independent MCMC initializations, which run over 10 million iterations. An increase in the number of clusters (=migration events) revealed a favoured two-cluster model for (b). Dataset (b) revealed highest support for a two-cluster model when one migration was allowed. Due to a putative lack of geographic structure in (c), an increase of migration events resulted in a messy looking plot and contradictory statistical support for different scenarios (i.e. increasing the number of migration events resulted in a shift in the support for clusters, that didn’t make any sense in a phylogeographic context).

### Morphometric analyses

Specimens were embedded in lactic acid for temporary slides and measurements were done using a compound light microscope (Olympus BH-2) and ocular micrometer. A set of 15 continuous variables (Supplementary Figure 5) was measured in 75 *Alismobates reticulatus* specimens from five different populations originating from three different Yaeyama islands (Yonaguni-jima, Iriomote-jima and Ishigaki-jima), and in 184 *Fortuynia* specimens from nine populations originating from six different islands (Iriomote-jima, Ishigaki-jima, Okinawa-jima, Amami-Ohshima, Omishima and Kii peninsula). Specimens used for morphometric comparison were not the same as used for molecular genetic analyses but they belonged to the same populations.

For univariate statistics of *Fortuynia*, minimum, maximum, mean and standard deviation for each variable were calculated. Multivariate analyses investigating differences between putative *Fortuynia* species included a Principal Component Analysis (PCA; using a variance-covariance matrix) and a Non-metric Multidimensional Scaling (NMDS; based on Euclidian distances, two-dimensional); both analyses were performed on log_10_-transformed size-corrected data. No rotation was applied to the multivariate data. Size correction was done by dividing each variable through the geometric mean of the respective specimen.

Kruskal-Wallis and a Mann-Whitney U tests (Bonferroni corrected p-values) were used to compare the means of variables for pairwise comparisons in order to clarify if single variables differ significantly among populations of *Fortuynia* and *Alismobates*. Additionally, a Discriminant Analysis (LDA) was performed on log_10_-transformed raw data to show size and shape differences between populations. As a slight sexual dimorphism is present in the studied species, the same analysis was repeated with males and females separated to obtain clearer results (except for *F. shibai*, where sample size was insufficient). All analyses were performed with PAST 3.11^57^.

### Drawings and photographs

Preserved animals were embedded in Berlese mountant for microscopic investigation in transmitted light. Drawings were made with an Olympus BH-2 Microscope equipped with a drawing attachment. These drawings were first scanned, then processed and digitized with the free and open-source vector graphics editor Inkscape 0.92 (https://inkscape.org).

For photographic documentation, specimens were air-dried and photographed with a Keyence VHX-5000 digital microscope.

Morphological terminology used in this paper follows^10,58–60^.

## Supplementary information

Supplementary information

## Data Availability

The genetic datasets generated during and/or analyzed during the current study are available in the GenBank repository, [https://www.ncbi.nlm.nih.gov/genbank/]. The morphometric datasets generated during and/or analyzed during the current study are available from the corresponding author on reasonable request.
