# Author Correction: Phylogeographic patterns of intertidal arthropods (Acari, Oribatida) from southern Japanese islands reflect paleoclimatic events

**DOI:** 10.1038/s41598-020-60883-w

**Published:** 2020-03-04

**Authors:** Tobias Pfingstl, Maximilian Wagner, Shimpei F. Hiruta, Stephan Koblmüller, Wataru Hagino, Satoshi Shimano

**Affiliations:** 1 Institute of Biology, University of Graz, Universitätsplatz 2, 8010 Graz, Austria; 2 Center for Molecular Biodiversity Research, National Museum of Nature and Science, Amakubo 4-1-1, Tsukuba, Ibaraki 305-0005 Japan; 3 Department of Bioresource Engineering, National Institute of Technology, Okinawa College, Henoko 905, Nago-City, Okinawa 905-2192 Japan; 4 Science Research Center, Hosei University, Fujimi 2-17-1 Chiyoda-ku, Tokyo, 102-8160 Japan

Correction to: *Scientific Reports* 10.1038/s41598-019-55270-z, published online 13 December 2019

The original version of this Article did not meet the requirement of the amendment of Article 8.5.3 of the International Code of Zoological Nomenclature^1^. To fully meet the amended provisions of the Code, the following Nomenclatural Acts have been added to the Materials and Methods section:

**Nomenclatural Acts**.

The electronic edition of this article conforms to the requirements of the amended International Code of Zoological Nomenclature, and hence the new names contained herein are available under that Code from the electronic edition of this article. This published work and the nomenclatural acts it contains have been registered in ZooBank, the online registration system for the ICZN. The ZooBank LSIDs (Life Science Identifiers) can be resolved and the associated information viewed through any standard web browser by appending the LSID to the prefix “http://zoobank.org/”. The LSID for this publication is: urn:lsid:zoobank.org:act:9A926270-7E55-4BD0-969D-B64C16646C5C

## References

[1] Amendment of Articles 8, 9, 10, 21 and 78 of the International Code of Zoological Nomenclature to expand and refine methods of publication. ZooKeys **219**, 1–10, 10.3897/zookeys.219.3944 (2012). 10.3897/zookeys.219.3994 PMC3433695 22977348

