# Supplementary material for: Phylogeographic patterns of intertidal arthropods (Acari, Oribatida) from southern Japanese islands reflect paleoclimatic events
Source: Sci Rep. 2019 Dec 13;9:19042. doi: 10.1038/s41598-019-55270-z (PMC6911088; doi:10.1038/s41598-019-55270-z)
Supplement: Supplementary file 1 — Supplementary information [file 41598_2019_55270_MOESM1_ESM.pdf]

Phylogeographic patterns of intertidal arthropods (Acari, Oribatida) from southern Japanese islands reflecting the geological history.

Tobias Pfingstl, Maximilian Wagner, Shimpei F. Hiruta, Stephan Koblmüller, Wataru Hagino and Satoshi Shimano

Supplementary material

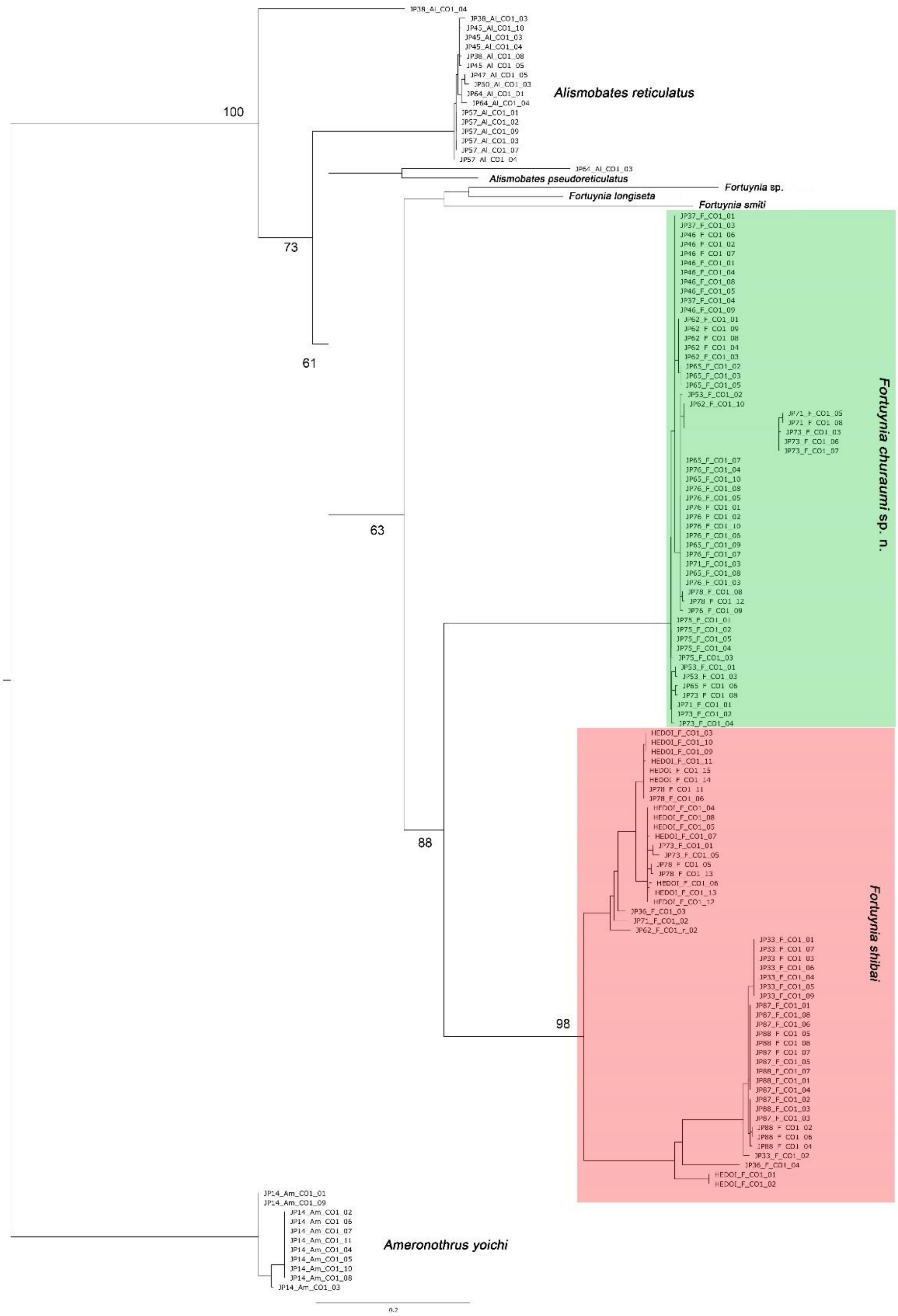

Supplementary Figure 1. IQ tree based on COI sequence data.

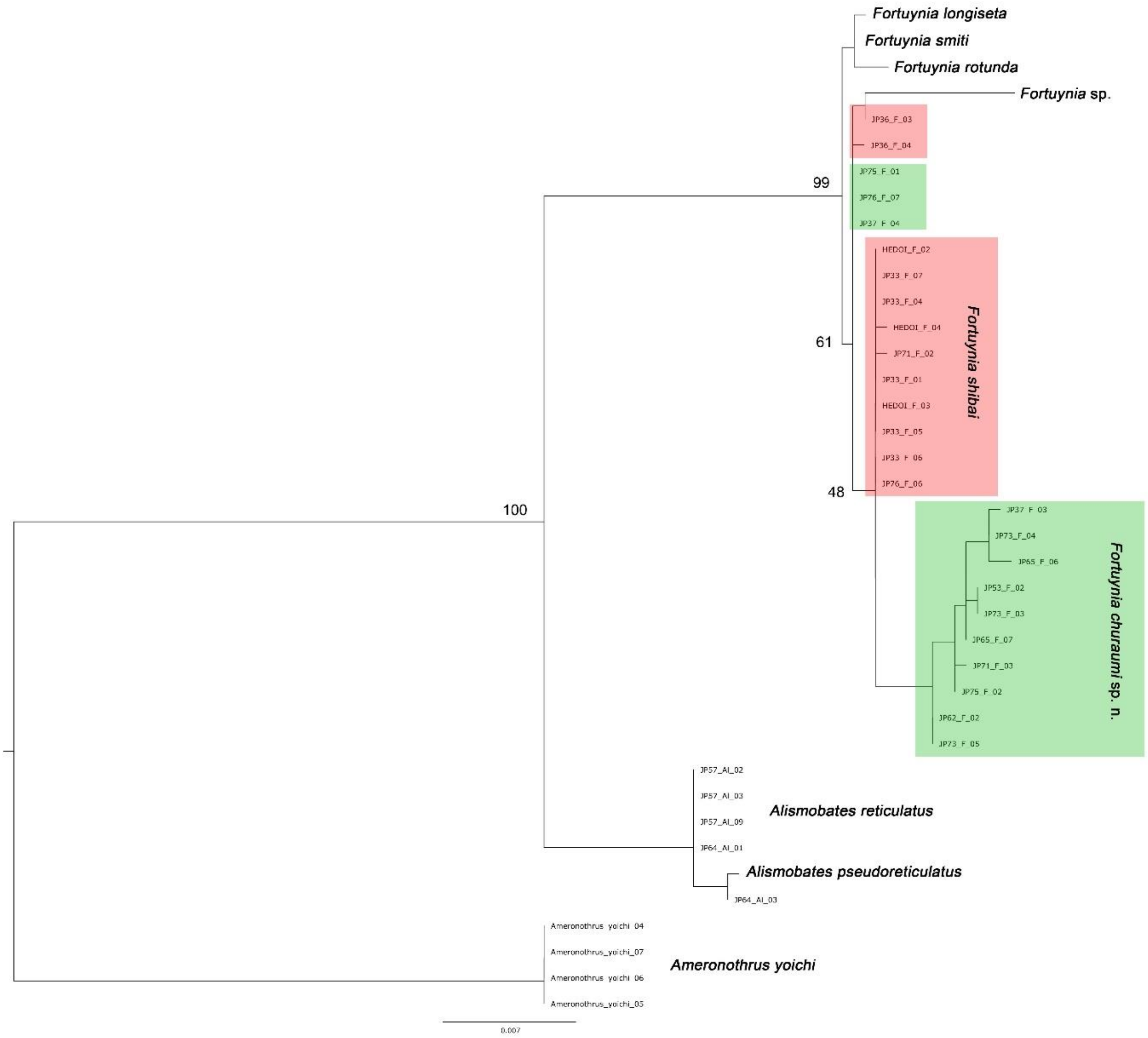

Supplementary Figure 2 IQ tree based on 18S rRNA sequence data.

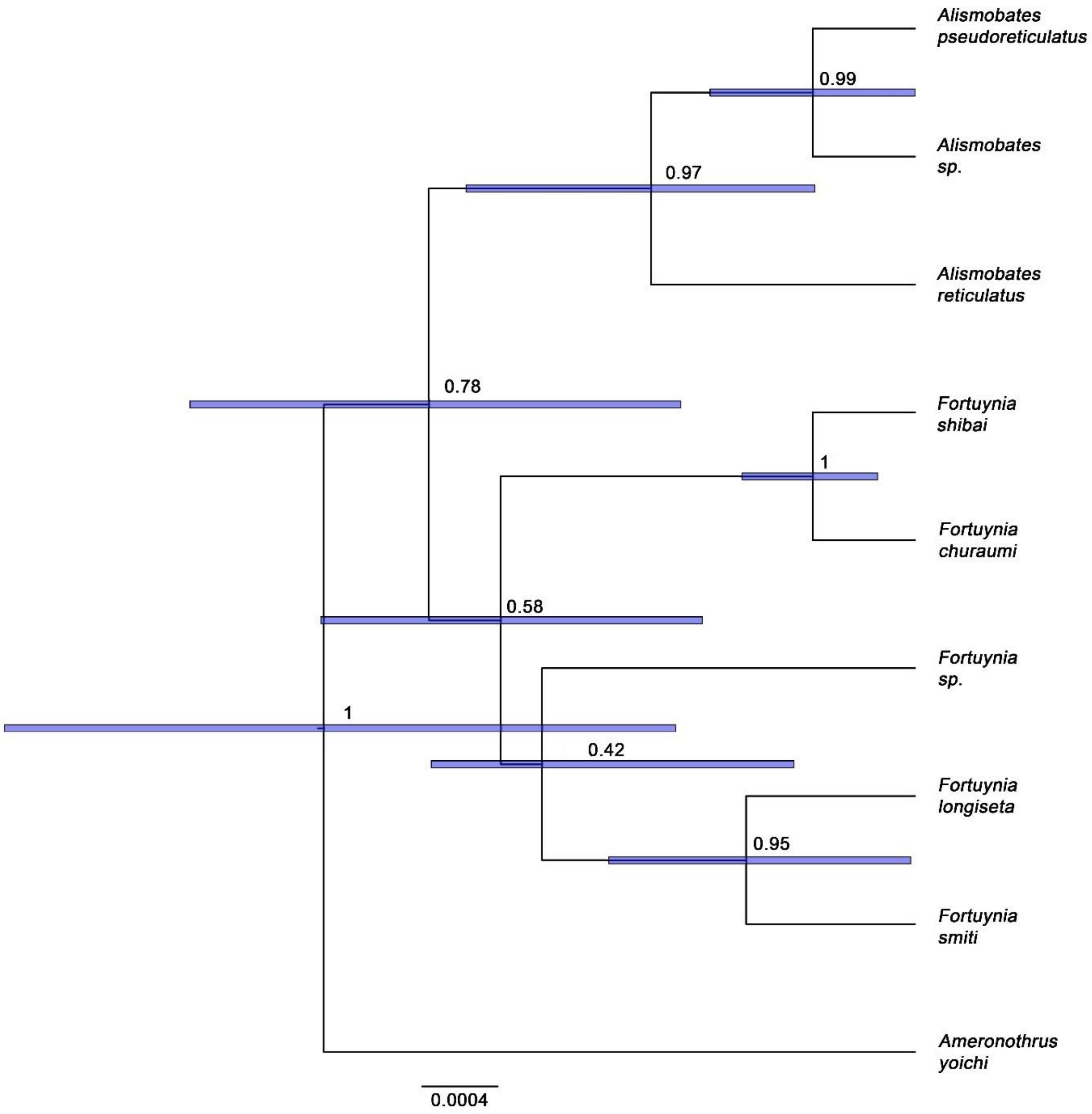

Supplementary Figure 3 Multispecies coalescent tree inferred in StarBeast2. Node bars indicate 95% highest posterior density (HPD) intervals of relative divergence time estimates.

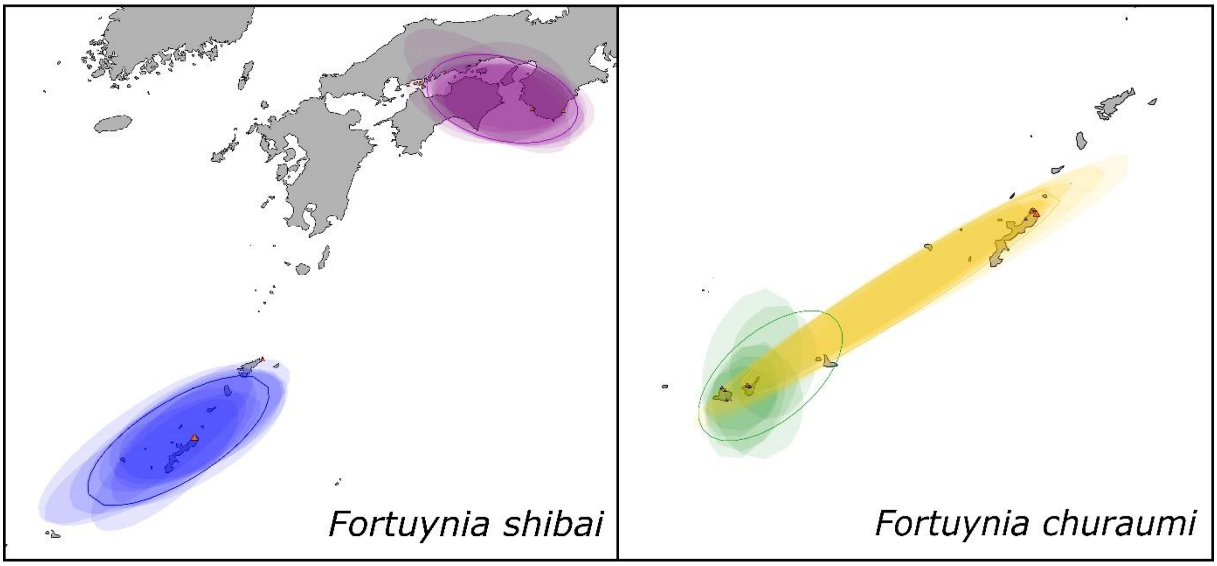

Supplementary Figure 4 Bayesian phylogeographical and ecological clustering (BPEC) analyses for the COI datasets of *Fortuynia shibai* and *F. churaumi* sp. n. Phylogeographic clusters are represented by colored contour plots which are placed at the "center" of each population cluster. Shaded ellipses represent the radius of 50% concentration contours around the cluster and solid lines the posterior means. Orange triangles show sampling locations; larger triangles correspond to most likely ancestral locations. This figure was generated with the R-package "bayesian phylogeographic and ecological clustering" (BPEC)[54]

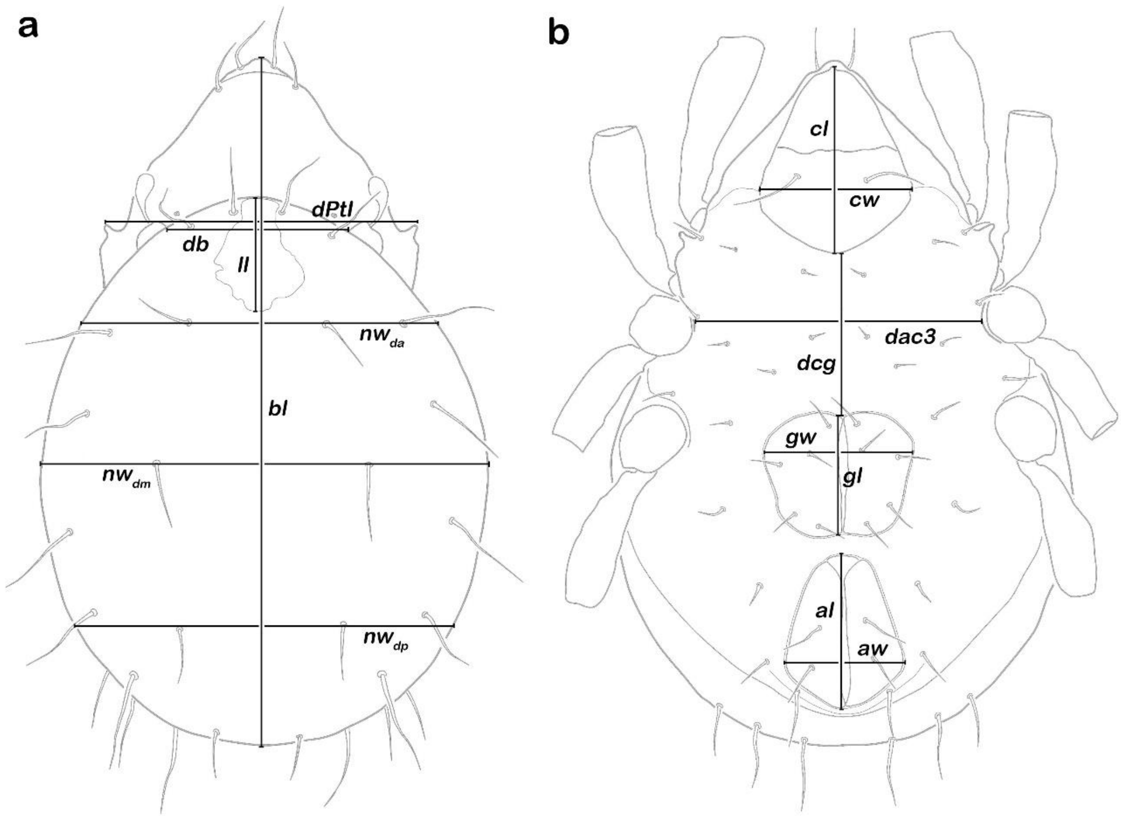

Supplementary Figure 5 Graphic illustration of measured continuous variables shown on a simplified drawing of *Fortuynia* (the same variables apply to *Alismobates*). (a) - dorsal aspect: *bl* body length, *dPtI* distance between pedotecta 1, *db* distance between bothridia, *ll* lenticulus length, nw_{da} notogastral width on level of seta *da*, nw_{dm} notogastral width on level of seta *dm*, nw_{dp} notogastral width on level of seta *dp*. (b) - ventral aspect: *cl* camerostome length, *cw* camerostome width, *dcg* distance between camerostome and genital orifice, *dac3* distance between acetabula 3, *gl* genital orifice length, *gw* genital orifice width, *al* anal orifice length, *aw* anal orifice width

Supplementary Table 1 Loadings of the first three principal components gained from PCA on size corrected data of two intertidal *Fortuynia* species.

	PC 1	PC 2	PC 3
bl	0.050135	-0.019548	0.063854
dPtI	-0.14038	-0.062033	-0.09712
db	-0.37079	-0.10851	-0.0524
ll	0.059937	0.8892	-0.28676
nw_{da}	-0.17003	0.18559	0.4954
nw_{dm}	-0.021685	-0.065662	0.096542
nw_{dp}	0.15537	-0.3369	-0.34092
cl	-0.10157	-0.13098	-0.28226
cw	-0.14602	-0.071217	-0.094722
dcg	0.01686	-0.083775	-0.40669
dac3	-0.14455	0.0015476	0.010169
gl	0.68829	-0.063967	0.027625
gw	0.43531	-0.022275	0.37011
al	-0.2524	-0.032843	0.3352
aw	-0.058469	-0.078393	0.16217

Supplementary Table 2. Univariate statistics for five different *Fortuynia shibai* populations from four Japanese islands (the two populations from the Kii peninsula were pooled for this analysis). Minimum–maximum (mean±standard deviation) of each measured variable given in µm. KW – Kruskal-Wallis Test, *0.01 < p < 0.05, **0.001< p < 0.01, ***p < 0.001; MWU – Mann-Whitney U test, - = no significant difference, letter indicates significant difference, a = Okinawa vs. Kii-hantō, b = Amami vs. Okinawa.

variable	Ishigaki JP_62	Amami JP_78	Omishima JP_33	Okinawa Hedo I	Kii JP_87 / 88	KW	MWU
bl	469–506 (492±19.9)	496–525 (499±18.4)	513	478–525 (507±11.1)	469–525 (501±11.9)	*	-
dPtI	200–212 (207±6.2)	197–219 (212±6.2)	212–222 (217±7.1)	203–222 (214±4)	203–215 (211±3.7)	*	-
db	105–120 (113±7.5)	108–120 (115±3.4)	120–123 (122±2.1)	99–123 (114±5)	114–126 (119±4)	***	a
ll	77–105 (86±16.2)	80–117 (97±13.1)	86–108 (97±15.6)	80–117 (102±11.3)	74–105 (92±6.1)	*	a
nw_{da}	240–271 (254±15.6)	237–395 (265±17)	302–305 (304±2.1)	234–292 (260±16)	222–302 (272±17.3)	***	a
nw_{dm}	295–312 (305±8.7)	292–338 (314±14.3)	331–344 (338±9.2)	295–331 (316±10.4)	299–338 (316±9.5)	-	-
nw_{dp}	240–262 (252±11.2)	210–292 (263±22.4)	277	237–286 (263±14.4)	231–292 (258±14.8)	-	-
cl	135–142 (139±3.5)	120–148 (138±6.9)	129–145 (137±11.3)	129–150 (138±5)	132–154 (139±4.8)	-	-
cw	95–108 (103±6.8)	102–108 (105±2.7)	102–111 (107±6.4)	102–111 (107±2.3)	102–111 (107±1.9)	-	-
dcg	102–108 (105±3)	99–117 (108±5.9)	105–120 (113±10.6)	92–114 (109±4.4)	99–120 (109±5.6)	-	-
dac3	179–188 (182±4.9)	175–191 (183±5.5)	185–197 (191±8.5)	163–194 (185±5.8)	185–200 (194±3.8)	***	a, b
gl	92–117 (109±14.4)	83–120 (107±12.1)	92–105 (99±9.2)	99–123 (114±7.2)	91–114 (99±5.7)	***	a
gw	111–139 (128±15.1)	111–142 (127±11.3)	126–129 (128±2.1)	117–142 (134±7.2)	105–139 (119±7.5)	***	a, b
al	102–111 (106±4.6)	95–114 (105±6.1)	99–114 (107±10.6)	92–111 (101±5.2)	92–120 (108±5.7)	***	a
aw	80–92 (87±6.2)	80–89 (85±3.3)	89–92 (91±2.1)	80–92 (87±3.4)	80–94 (86±3.4)	-	-

Supplementary Table 3. Univariate statistics for seven different *Fortuynia churaumi* sp. n. populations from four Japanese islands. Minimum–maximum (mean±standard deviation) of each measured variable given in µm. KW – Kruskal-Wallis Test, *0.01 < p < 0.05, ** 0.001< p < 0.01, *** p < 0.001.

	Iriomote	Ishigaki		Okinawa			Amami	
**variable**	JP_46	JP_62	JP_65	JP_72	JP_73	JP_76	JP_78	KW
bl	413–444 (427±9.8)	400–431 (420±9.8)	388–425 (411±9.6)	394–425 (410±11.1)	400–431 (417±10.2)	379–425 (406±13)	406–425 (416±7.7)	***
dPtI	188–197 (191±2.6)	182–194 (189±3.4)	179–197 (187±4.6)	182–191 (185±2.7)	185–195 (190±4)	175–185 (182±3.2)	185–194 (189±2.9)	***
db	105–117 (111±4.2)	105–117 (111±3.8)	99–111 (108±3.1)	102–111 (107–2.8)	102–114 (109±4.2)	102–111 (107±3.1)	105–114 (111±3.5)	**
ll	71–92 (85±6.5)	77–92 (86±5.8)	65–92 (80±8)	74–85 (78±3.3)	71–89 (79±5.6)	63–86 (77±5.7)	74–93 (83±7.2)	**
nw_{da}	215–259 (240±12)	209–249 (236±12)	206–246 (227±10.5)	200–243 (226±10.3)	222–255 (239±10.7)	203–246 (227±11.7)	215–243 (229±10)	**
nw_{dm}	259–283 (275±6.4)	255–283 (274±8.5)	249–277 (263±8.2)	252–280 (266±7)	249–286 (272±11.1)	249–274 (262±9.6)	262–283 (271±7)	***
nw_{dp}	182–228 (213±12)	197–234 (217±13)	185–237 (210±14.3)	191–243 (217±12.7)	185–252 (216±21)	182–225 (208±10.8)	203–234 (216±12)	-
cl	117–126(120±3)	117–123 (122±2.4)	114–123 (120±2.7)	114–126 (120±3.2)	114–132 (121±6)	114–123 (117±2.7)	117–126 (121±3.3)	**
cw	92–99 (96±2.3)	89–95 (93±1.9)	92–99 (93±1.9)	89–95 (93±2)	89–99 (93±3.2)	89–95 (92±1.7)	92–96 (94±1.6)	***
dcg	86–95 (90±2.5)	86–92 (88±2.3)	86–95 (91±2.7)	86–92 (90±1.8)	86–92 (90±2.5)	83–92 (87±3.1)	83–95 (90±4.5)	**
dac3	151–182 (172±7.5)	159–175 (168±5)	154–169 (162±4.2)	151–163 (159±2.7)	155–166 (161±3.8)	148–163 (155±4)	157–166 (162±2.7)	***
gl	68–86 (76±4.1)	71–83 (77±3.8)	68–83 (74±4.4)	68–83 (74±5)	65–83 (75±5.2)	65–80 (74±4.6)	71–80 (76±3)	-
gw	92–105 (100±4.5)	89–105 (99±5.5)	86–105 (95±6.3)	86–102 (95±5.5)	86–108 (98±7.4)	86–102 (94±5.3)	89–105 (98±5.6)	*
al	89–102 (97±3.8)	89–99 (94±3.1)	86–99 (92±3)	86–95 (93±2.6)	92–102 (94±3.2)	86–99 (92±4.4)	86–99 (91±3.7)	***
aw	74–82 (77±2.6)	68–80 (74±3.6)	68–77 (74±3.3)	68–77 (74±2.9)	71–80 (75±2.8)	68–77 (73±2.8)	71–80 (75±2.6)	**

Supplementary Table 4. Univariate statistics for four different *Alismobates reticulatus* populations from the islands Iriomote-jima, Yonaguni-jima and Ishigaki-jima. Minimum–maximum (mean±standard deviation) of each measured variable given in µm. KW – Kruskal-Wallis Test, *0.01 < p < 0.05, **0.001< p < 0.01, ***p < 0.001. MWU – Mann-Whitney U test, - = no significant difference, letter indicates significant difference, a = JP_38 vs. JP_91, b = JP_45 vs. JP_91, c = JP_47 vs. JP_91, d = JP_57 vs. JP_91, e = JP_38 vs. JP_57, f = JP_45 vs. JP_57, g = JP_38 vs. JP_47, h = JP_45 vs. JP_47, i = JP_38 vs. JP_45

	Iriomote			Yonaguni	Ishigaki		
variable	JP_38	JP_45	JP_47	JP_57	JP_91	KW	MWU
bl	277–308 (292±9.4)	283–313 (297±7.9)	271–299 (285±9.6)	274–308 (292±7.4)	289–319 (306±8.9)	***	a, b, c, d
dPtI	123–132 (128±2.6)	123–132 (128±2.3)	120–129 (124±3.6)	120–126 (124±1.7)	123–135 (129±3.1)	***	d, e, f
db	71–80 (75±2.2)	71–77 (74±1.8)	68–74 (70±2.1)	71–74 (72±1.5)	74–83 (77±2.2)	***	b, e, g, h
ll	49–65 (57±5.1)	43–68 (55±6.3)	52–62 (58±4.6)	46–59 (52±5)	46–74 (59±7.8)	*	-
nw_{da}	163–194 (179±8.1)	170–196 (182±7.3)	160–185 (172±8.9)	154–194 (175±10.3)	169–206 (193±11.3)	***	a, b, c, d
nw_{dm}	185–219 (203±8.9)	197–219 (206±6.3)	188–206 (197±6.4)	185–212 (200±7.7)	200–222 (212±5.8)	***	c, h, d
nw_{dp}	166–212 (183±13)	170–203 (186±8.5)	163–183 (174±7.8)	166–191 (180±8)	170–203 (186±9.2)	*	-
cl	77–89 (84±3.3)	77–89 (83±3.2)	77–89 (82±3.8)	77–86 (81±2.6)	77–86 (83±3.1)	-	-
cw	62–65 (64±1.3)	59–65 (62±1.3)	59–65 (63±2.3)	59–65 (62±1.8)	59–68 (63±2.6)	*	i
dcg	71–77 (74±2.1)	68–77 (72±2.1)	65–77 (72±3.8)	68–77 (73±2.6)	71–80 (74±2.9)	-	-
dac3	106–117 (111±3.1)	105–111 (109±1.8)	99–114 (106±4.8)	102–110 (106±2.3)	108–117 (112±2.8)	***	d, e, f
gl	43–57 (49±4.2)	46–55 (50±2.8)	43–52 (48±2.9)	43–55 (47±3)	43–55 (51±3.1)	**	d, f
gw	55–68 (61±4.4)	55–68 (63±4.1)	54–65 (57±3.9)	55–65 (60±3.6)	55–68 (63±3.6)	**	-
al	59–68 (63±2.2)	59–68 (64±2)	59–62 (61±1.1)	55–65 (62±2.7)	62–68 (65±2.4)	***	c, d
aw	48–55 (52±2)	49–55 (53±1.9)	46–55 (50±2.7)	47–52 (50±1.6)	50–52 (52±0.8)	***	c, d, f

Supplementary Table 5. PCR and cycle sequencing primers used in this study. Annealing temperatures are given in °C. W = A or T; Y = C or T; R = A or G; D = A G or T; I = inosine.

Region	Primer	Sequence	Annealing temp	source
**COI 2**	Mite COI - 2F	TTYGAYCCIDYIGGRGGAGGAGATCC	1st 46	Otto & Wilson 2001
	Mite COI - 2R	GGRTARTCWGARTAWCGNCGWGGTAT	2nd 48	
**18S PCR**	18Sfw	CTTGTCTCAAAGATTAAGCCATGCA	47	Dabert et al. 2010
	rev18S	TGATCCTTCCGCAGGTTCACCT		
18S Cycle	*fw390**	*AATCAGGGTTCGATTCCGGAGA*		Dabert et al. 2010
	*rev480**	*GTTATTTTTCGTCACTACCT*		
	*fw770**	*ACTTTGAAAAAATTAGAGTGC*		
	*fw1230**	*TGAAACTTAAAGGAATTGACG*		Skoracka & Dabert 2010

* internal cycle sequencing primers

Supplementary Table 6. Sample IDs, coordinates and GenBank accession numbers for *COI*, and *18S* sequences comprising all specimens included in genetic investigations. Sequences generated in this study appear in bold.

Field_ID	Locality	Coordinates	COI	18S	species	source
JP38_Al_03	Iriomote Island	24°19'17.43"N 123°54'38.87"E	MN385052		*A. reticulatus*	this study
JP38_Al_08	Iriomote Island	24°19'17.43"N 123°54'38.87"E	MN385053		*A. reticulatus*	this study
JP45_Al_03	Iriomote Island	24°16'32.38"N 123°52'58.87"E	MN385054		*A. reticulatus*	this study
JP45_Al_04	Iriomote Island	24°16'32.38"N 123°52'58.87"E	MN385055		*A. reticulatus*	this study
JP45_Al_05	Iriomote Island	24°16'32.38"N 123°52'58.87"E	MN385056		*A. reticulatus*	this study
JP45_Al_10	Iriomote Island	24°16'32.38"N 123°52'58.87"E	MN385057		*A. reticulatus*	this study
JP47_Al_05	Iriomote Island	24°23'52.93"N 123°49'42.72"E	MN385066		*A. reticulatus*	this study
JP50_Al_03	Iriomote Island	24°23'49.18"N 123°49'19.59"E	MN385067		*A. reticulatus*	this study
JP57_Al_01	Yonaguni Island	24°26'25.78"N 122°58'27.44"E	MN385071		*A. reticulatus*	this study
JP57_Al_02	Yonaguni Island	24°26'25.78"N 122°58'27.44"E	MN385072	MN372417	*A. reticulatus*	this study
JP57_Al_03	Yonaguni Island	24°26'25.78"N 122°58'27.44"E	MN385073	MN372418	*A. reticulatus*	this study
JP57_Al_04	Yonaguni Island	24°26'25.78"N 122°58'27.44"E	MN385074		*A. reticulatus*	this study
JP57_Al_07	Yonaguni Island	24°26'25.78"N 122°58'27.44"E	MN385075		*A. reticulatus*	this study
JP57_Al_09	Yonaguni Island	24°26'25.78"N 122°58'27.44"E	MN385076	MN372419	*A. reticulatus*	this study
JP64_Al_01	Ishigaki Island	24°26'33.63"N 124° 8'29.79"E	MN385084	MN372421	*A. reticulatus*	this study
JP64_Al_04	Ishigaki Island	24°26'33.63"N 124° 8'29.79"E	MN385086		*A. reticulatus*	this study
JP64_Al_03	Ishigaki Island	24°26'33.63"N 124° 8'29.79"E	MN385085	MN372422	*Alismobates* sp.	this study
JP37_F_01	Iriomote Island	24°26'11.22"N 123°46'36.80"E	MN385049		*F. churaumi* sp. n.	this study
JP37_F_03	Iriomote Island	24°26'11.22"N 123°46'36.80"E	MN385050	MN372414	*F. churaumi* sp. n.	this study
JP37_F_04	Iriomote Island	24°26'11.22"N 123°46'36.80"E	MN385051	MN372415	*F. churaumi* sp. n.	this study
JP46_F_01	Iriomote Island	24°16'0.02"N 123°50'45.71"E	MN385058		*F. churaumi* sp. n.	this study
JP46_F_02	Iriomote Island	24°16'0.02"N 123°50'45.71"E	MN385059		*F. churaumi* sp. n.	this study
JP46_F_04	Iriomote Island	24°16'0.02"N 123°50'45.71"E	MN385060		*F. churaumi* sp. n.	this study

JP46_F_05	Iriomote Island	24°16'0.02"N 123°50'45.71"E	MN385061		*F. churaumi* sp. n.	this study
JP46_F_06	Iriomote Island	24°16'0.02"N 123°50'45.71"E	MN385062		*F. churaumi* sp. n.	this study
JP46_F_07	Iriomote Island	24°16'0.02"N 123°50'45.71"E	MN385063		*F. churaumi* sp. n.	this study
JP46_F_08	Iriomote Island	24°16'0.02"N 123°50'45.71"E	MN385064		*F. churaumi* sp. n.	this study
JP46_F_09	Iriomote Island	24°16'0.02"N 123°50'45.71"E	MN385065		*F. churaumi* sp. n.	this study
JP53_F_01	Iriomote Island	24°23'53.48"N 123°49'20.21"E	MN385068		*F. churaumi* sp. n.	this study
JP53_F_02	Iriomote Island	24°23'53.48"N 123°49'20.21"E	MN385069	MN372416	*F. churaumi* sp. n.	this study
JP53_F_03	Iriomote Island	24°23'53.48"N 123°49'20.21"E	MN385070		*F. churaumi* sp. n.	this study
JP62_F_01	Ishigaki Island	24°27'49.14"N 124° 8'39.09"E	MN385077		*F. churaumi* sp. n.	this study
JP62_F_03	Ishigaki Island	24°27'49.14"N 124° 8'39.09"E	MN385079		*F. churaumi* sp. n.	this study
JP62_F_04	Ishigaki Island	24°27'49.14"N 124° 8'39.09"E	MN385080		*F. churaumi* sp. n.	this study
JP62_F_08	Ishigaki Island	24°27'49.14"N 124° 8'39.09"E	MN385081		*F. churaumi* sp. n.	this study
JP62_F_09	Ishigaki Island	24°27'49.14"N 124° 8'39.09"E	MN385082		*F. churaumi* sp. n.	this study
JP62_F_10	Ishigaki Island	24°27'49.14"N 124° 8'39.09"E	MN385083		*F. churaumi* sp. n.	this study
JP65_F_02	Ishigaki Island	24°26'56.11"N 124°10'46.41"E	MN385087		*F. churaumi* sp. n.	this study
JP65_F_03	Ishigaki Island	24°26'56.11"N 124°10'46.41"E	MN385088		*F. churaumi* sp. n.	this study
JP65_F_05	Ishigaki Island	24°26'56.11"N 124°10'46.41"E	MN385089		*F. churaumi* sp. n.	this study
JP65_F_06	Ishigaki Island	24°26'56.11"N 124°10'46.41"E	MN385090	MN372423	*F. churaumi* sp. n.	this study
JP65_F_07	Ishigaki Island	24°26'56.11"N 124°10'46.41"E	MN385091	MN372424	*F. churaumi* sp. n.	this study
JP65_F_08	Ishigaki Island	24°26'56.11"N 124°10'46.41"E	MN385092		*F. churaumi* sp. n.	this study
JP65_F_09	Ishigaki Island	24°26'56.11"N 124°10'46.41"E	MN385093		*F. churaumi* sp. n.	this study
JP65_F_10	Ishigaki Island	24°26'56.11"N 124°10'46.41"E	MN385094		*F. churaumi* sp. n.	this study
JP71_F_01	Okinawa Island	26°50'44.60"N 128°17'22.00"E	MN385095		*F. churaumi* sp. n.	this study
JP71_F_03	Okinawa Island	26°50'44.60"N 128°17'22.00"E	MN385097	MN372426	*F. churaumi* sp. n.	this study
JP71_F_05	Okinawa Island	26°50'44.60"N 128°17'22.00"E	MN385098		*F. churaumi* sp. n.	this study
JP71_F_08	Okinawa Island	26°50'44.60"N 128°17'22.00"E	MN385099		*F. churaumi* sp. n.	this study

JP73_F_02	Okinawa Island	26°49'50.91"N 128°14'43.35"E	MN385101		*F. churaumi* sp. n.	this study
JP73_F_03	Okinawa Island	26°49'50.91"N 128°14'43.35"E	MN385102	MN372427	*F. churaumi* sp. n.	this study
JP73_F_04	Okinawa Island	26°49'50.91"N 128°14'43.35"E	MN385103	MN372428	*F. churaumi* sp. n.	this study
JP73_F_06	Okinawa Island	26°49'50.91"N 128°14'43.35"E	MN385105		*F. churaumi* sp. n.	this study
JP73_F_07	Okinawa Island	26°49'50.91"N 128°14'43.35"E	MN385106		*F. churaumi* sp. n.	this study
JP73_F_08	Okinawa Island	26°49'50.91"N 128°14'43.35"E	MN385107		*F. churaumi* sp. n.	this study
JP75_F_01	Okinawa Island	26°43'47.66"N 128° 9'38.15"E	MN385108	MN372430	*F. churaumi* sp. n.	this study
JP75_F_02	Okinawa Island	26°43'47.66"N 128° 9'38.15"E	MN385109	MN372431	*F. churaumi* sp. n.	this study
JP75_F_03	Okinawa Island	26°43'47.66"N 128° 9'38.15"E	MN385110		*F. churaumi* sp. n.	this study
JP75_F_04	Okinawa Island	26°43'47.66"N 128° 9'38.15"E	MN385111		*F. churaumi* sp. n.	this study
JP75_F_05	Okinawa Island	26°43'47.66"N 128° 9'38.15"E	MN385112		*F. churaumi* sp. n.	this study
JP76_F_01	Okinawa Island	26°47'40.24"N 128°19'7.65"E	MN385113		*F. churaumi* sp. n.	this study
JP76_F_02	Okinawa Island	26°47'40.24"N 128°19'7.65"E	MN385114		*F. churaumi* sp. n.	this study
JP76_F_03	Okinawa Island	26°47'40.24"N 128°19'7.65"E	MN385115		*F. churaumi* sp. n.	this study
JP76_F_04	Okinawa Island	26°47'40.24"N 128°19'7.65"E	MN385116		*F. churaumi* sp. n.	this study
JP76_F_05	Okinawa Island	26°47'40.24"N 128°19'7.65"E	MN385117		*F. churaumi* sp. n.	this study
JP76_F_06	Okinawa Island	26°47'40.24"N 128°19'7.65"E	MN385118	MN372432	*F. churaumi* sp. n.	this study
JP76_F_07	Okinawa Island	26°47'40.24"N 128°19'7.65"E	MN385119	MN372433	*F. churaumi* sp. n.	this study
JP76_F_08	Okinawa Island	26°47'40.24"N 128°19'7.65"E	MN385120		*F. churaumi* sp. n.	this study
JP76_F_09	Okinawa Island	26°47'40.24"N 128°19'7.65"E	MN385121		*F. churaumi* sp. n.	this study
JP76_F_10	Okinawa Island	26°47'40.24"N 128°19'7.65"E	MN385122		*F. churaumi* sp. n.	this study
JP78_F_08	Amami Island	28°28'23.36"N 129°43'8.03"E	MN385125		*F. churaumi* sp. n.	this study
JP78_F_12	Amami Island	28°28'23.36"N 129°43'8.03"E	MN385127		*F. churaumi* sp. n.	this study
HEDOI_F_01	Okinawa Island	26°51'49.72"N 128°15'57.31"E	MN385024		*F. shibai*	this study
HEDOI_F_02	Okinawa Island	26°51'49.72"N 128°15'57.31"E	MN385025	MN372404	*F. shibai*	this study
HEDOI_F_03	Okinawa Island	26°51'49.72"N 128°15'57.31"E	MN385026	MN372405	*F. shibai*	this study

HEDOI_F_04	Okinawa Island	26°51'49.72"N 128°15'57.31"E	MN385027	MN372406	*F. shibai*	this study
HEDOI_F_05	Okinawa Island	26°51'49.72"N 128°15'57.31"E	MN385028		*F. shibai*	this study
HEDOI_F_06	Okinawa Island	26°51'49.72"N 128°15'57.31"E	MN385029		*F. shibai*	this study
HEDOI_F_07	Okinawa Island	26°51'49.72"N 128°15'57.31"E	MN385030		*F. shibai*	this study
HEDOI_F_08	Okinawa Island	26°51'49.72"N 128°15'57.31"E	MN385031		*F. shibai*	this study
HEDOI_F_09	Okinawa Island	26°51'49.72"N 128°15'57.31"E	MN385032		*F. shibai*	this study
HEDOI_F_10	Okinawa Island	26°51'49.72"N 128°15'57.31"E	MN385033		*F. shibai*	this study
HEDOI_F_11	Okinawa Island	26°51'49.72"N 128°15'57.31"E	MN385034		*F. shibai*	this study
HEDOI_F_12	Okinawa Island	26°51'49.72"N 128°15'57.31"E	MN385035		*F. shibai*	this study
HEDOI_F_13	Okinawa Island	26°51'49.72"N 128°15'57.31"E	MN385036		*F. shibai*	this study
HEDOI_F_14	Okinawa Island	26°51'49.72"N 128°15'57.31"E	MN385037		*F. shibai*	this study
HEDOI_F_15	Okinawa Island	26°51'49.72"N 128°15'57.31"E	MN385038		*F. shibai*	this study
JP33_F_01	Omishima Island	34°13'26.60"N 132°59'0.68"E	MN385039	MN372407	*F. shibai*	this study
JP33_F_02	Omishima Island	34°13'26.60"N 132°59'0.68"E	MN385040		*F. shibai*	this study
JP33_F_03	Omishima Island	34°13'26.60"N 132°59'0.68"E	MN385041		*F. shibai*	this study
JP33_F_04	Omishima Island	34°13'26.60"N 132°59'0.68"E	MN385042	MN372408	*F. shibai*	this study
JP33_F_05	Omishima Island	34°13'26.60"N 132°59'0.68"E	MN385043	MN372409	*F. shibai*	this study
JP33_F_06	Omishima Island	34°13'26.60"N 132°59'0.68"E	MN385044	MN372410	*F. shibai*	this study
JP33_F_07	Omishima Island	34°13'26.60"N 132°59'0.68"E	MN385045	MN372411	*F. shibai*	this study
JP33_F_09	Omishima Island	34°13'26.60"N 132°59'0.68"E	MN385046		*F. shibai*	this study
JP36_F_03	Ishigaki Island	24°20'40.61"N 124°12'5.58"E	MN385047	MN372412	*F. shibai*	this study
JP36_F_04	Ishigaki Island	24°20'40.61"N 124°12'5.58"E	MN385048	MN372413	*F. shibai*	this study
JP62_F_02	Ishigaki Island	24°27'49.14"N 124° 8'39.09"E	MN385078	MN372420	*F. shibai*	this study
JP71_F_02	Okinawa Island	26°50'44.60"N 128°17'22.00"E	MN385096	MN372425	*F. shibai*	this study
JP73_F_01	Okinawa Island	26°49'50.91"N 128°14'43.35"E	MN385100		*F. shibai*	this study
JP73_F_05	Okinawa Island	26°49'50.91"N 128°14'43.35"E	MN385104	MN372429	*F. shibai*	this study

JP78_F_05	Amami Island	28°28'23.36"N 129°43'8.03"E	MN385123		*F. shibai*	this study
JP78_F_06	Amami Island	28°28'23.36"N 129°43'8.03"E	MN385124		*F. shibai*	this study
JP78_F_11	Amami Island	28°28'23.36"N 129°43'8.03"E	MN385126		*F. shibai*	this study
JP78_F_13	Amami Island	28°28'23.36"N 129°43'8.03"E	MN385128		*F. shibai*	this study
JP87_F_01	Kii Peninsula	33°41'32.0"N 135°20'04.0"E	MN385129		*F. shibai*	this study
JP87_F_02	Kii Peninsula	33°41'32.0"N 135°20'04.0"E	MN385130		*F. shibai*	this study
JP87_F_03	Kii Peninsula	33°41'32.0"N 135°20'04.0"E	MN385131		*F. shibai*	this study
JP87_F_04	Kii Peninsula	33°41'32.0"N 135°20'04.0"E	MN385132		*F. shibai*	this study
JP87_F_05	Kii Peninsula	33°41'32.0"N 135°20'04.0"E	MN385133		*F. shibai*	this study
JP87_F_06	Kii Peninsula	33°41'32.0"N 135°20'04.0"E	MN385134		*F. shibai*	this study
JP87_F_07	Kii Peninsula	33°41'32.0"N 135°20'04.0"E	MN385135		*F. shibai*	this study
JP87_F_08	Kii Peninsula	33°41'32.0"N 135°20'04.0"E	MN385136		*F. shibai*	this study
JP88_F_01	Kii Peninsula	33°39'19.1"N 135°58'44.8"E	MN385137		*F. shibai*	this study
JP88_F_02	Kii Peninsula	33°39'19.1"N 135°58'44.8"E	MN385138		*F. shibai*	this study
JP88_F_03	Kii Peninsula	33°39'19.1"N 135°58'44.8"E	MN385139		*F. shibai*	this study
JP88_F_04	Kii Peninsula	33°39'19.1"N 135°58'44.8"E	MN385140		*F. shibai*	this study
JP88_F_05	Kii Peninsula	33°39'19.1"N 135°58'44.8"E	MN385141		*F. shibai*	this study
JP88_F_06	Kii Peninsula	33°39'19.1"N 135°58'44.8"E	MN385142		*F. shibai*	this study
JP88_F_07	Kii Peninsula	33°39'19.1"N 135°58'44.8"E	MN385143		*F. shibai*	this study
JP88_F_08	Kii Peninsula	33°39'19.1"N 135°58'44.8"E	MN385144		*F. shibai*	this study
JP14_A_01	Hokkaido Island	43°14′52′′N 140°42′35′′E	MK883430		*A. yoichi*	[60]
JP14_A_02	Hokkaido Island	43°14′52′′N 140°42′35′′E	MK883431		*A. yoichi*	[60]
JP14_A_03	Hokkaido Island	43°14′52′′N 140°42′35′′E	MK883432		*A. yoichi*	[60]
JP14_A_04	Hokkaido Island	43°14′52′′N 140°42′35′′E	MK883433	MK880170	*A. yoichi*	[60]
JP14_A_05	Hokkaido Island	43°14′52′′N 140°42′35′′E	MK883434	MK880171	*A. yoichi*	[60]
JP14_A_06	Hokkaido Island	43°14′52′′N 140°42′35′′E	MK883435	MK880172	*A. yoichi*	[60]

JP14_A_07	Hokkaido Island	43°14′52′′N 140°42′35′′E	MK883436	MK880173	*A. yoichi*	60
JP14_A_08	Hokkaido Island	43°14′52′′N 140°42′35′′E	MK883437		*A. yoichi*	60
JP14_A_09	Hokkaido Island	43°14′52′′N 140°42′35′′E	MK883438		*A. yoichi*	60
JP14_A_10	Hokkaido Island	43°14′52′′N 140°42′35′′E	MK883439		*A. yoichi*	60
JP14_A_11	Hokkaido Island	43°14′52′′N 140°42′35′′E	MK883440		*A. yoichi*	60
A_MY_07_7	Pantai Pasir Hitam	6°25′24.24′′N 99°47′15.81′′E	MH285613	MH285696	*A. pseudo -reticulatus*	16
F_TH_06_5	Nang Thong Beach	8°37′46.76′′N 98°14′35.95′′E	MH285636	MH285693	*F. longiseta*	16
F_MY_11_1N	Datai Bay	6°26′01.39′′N 99°41′03.17′′E	MH285628	MH285694	*F. smiti*	16
F_MY_07_8N	Pantai Pasir Hitam	6°25′24.24′′N 99°47′15.81′′E	MH285627	MH285695	*Fortuynia* sp.	16
Fortuynia rotunda	Okinawa Island	26°27'22.9"N 127°56'29.8"E		AB818525	*F. rotunda*	61
